# Exploring the nexus of nuclear receptors in hematological malignancies

**DOI:** 10.1007/s00018-023-05085-z

**Published:** 2024-02-09

**Authors:** Mukesh Kumar Manickasamy, Anjana Sajeev, Bandari BharathwajChetty, Mohammed S. Alqahtani, Mohamed Abbas, Mangala Hegde, Babu Santha Aswani, Mehdi Shakibaei, Gautam Sethi, Ajaikumar B. Kunnumakkara

**Affiliations:** 1 Cancer Biology Laboratory, Department of Biosciences and Bioengineering, Indian Institute of Technology Guwahati (IITG), Guwahati, Assam 781039 India; 2 Radiological Sciences Department, College of Applied Medical Sciences, King Khalid University, 61421 Abha, Saudi Arabia; 3 BioImaging Unit, Space Research Centre, University of Leicester, Michael Atiyah Building, Leicester, LE1 7RH UK; 4 Electrical Engineering Department, College of Engineering, King Khalid University, 61421 Abha, Saudi Arabia; 5 Chair of Vegetative Anatomy, Department of Human-Anatomy, Musculoskeletal Research Group and Tumor Biology, Institute of Anatomy, Ludwig-Maximilian-University, 80336 Munich, Germany; 6 Department of Pharmacology, Yong Loo Lin School of Medicine, National University of Singapore, Singapore, 117600 Singapore; 7 NUS Centre for Cancer Research (N2CR), Yong Loo Lin School of Medicine, National University of Singapore, Singapore, 117599 Singapore

**Keywords:** Hematological malignancies, Leukemia, Lymphoma, Multiple myeloma, Nuclear receptors, Differentiation, Apoptosis, Homeostasis

## Abstract

Hematological malignancies (HM) represent a subset of neoplasms affecting the blood, bone marrow, and lymphatic systems, categorized primarily into leukemia, lymphoma, and multiple myeloma. Their prognosis varies considerably, with a frequent risk of relapse despite ongoing treatments. While contemporary therapeutic strategies have extended overall patient survival, they do not offer cures for advanced stages and often lead to challenges such as acquisition of drug resistance, recurrence, and severe side effects. The need for innovative therapeutic targets is vital to elevate both survival rates and patients' quality of life. Recent research has pivoted towards nuclear receptors (NRs) due to their role in modulating tumor cell characteristics including uncontrolled proliferation, differentiation, apoptosis evasion, invasion and migration. Existing evidence emphasizes NRs' critical role in HM. The regulation of NR expression through agonists, antagonists, or selective modulators, contingent upon their levels, offers promising clinical implications in HM management. Moreover, several anticancer agents targeting NRs have been approved by the Food and Drug Administration (FDA). This review highlights the integral function of NRs in HM's pathophysiology and the potential benefits of therapeutically targeting these receptors, suggesting a prospective avenue for more efficient therapeutic interventions against HM.

**Graphical abstract:**

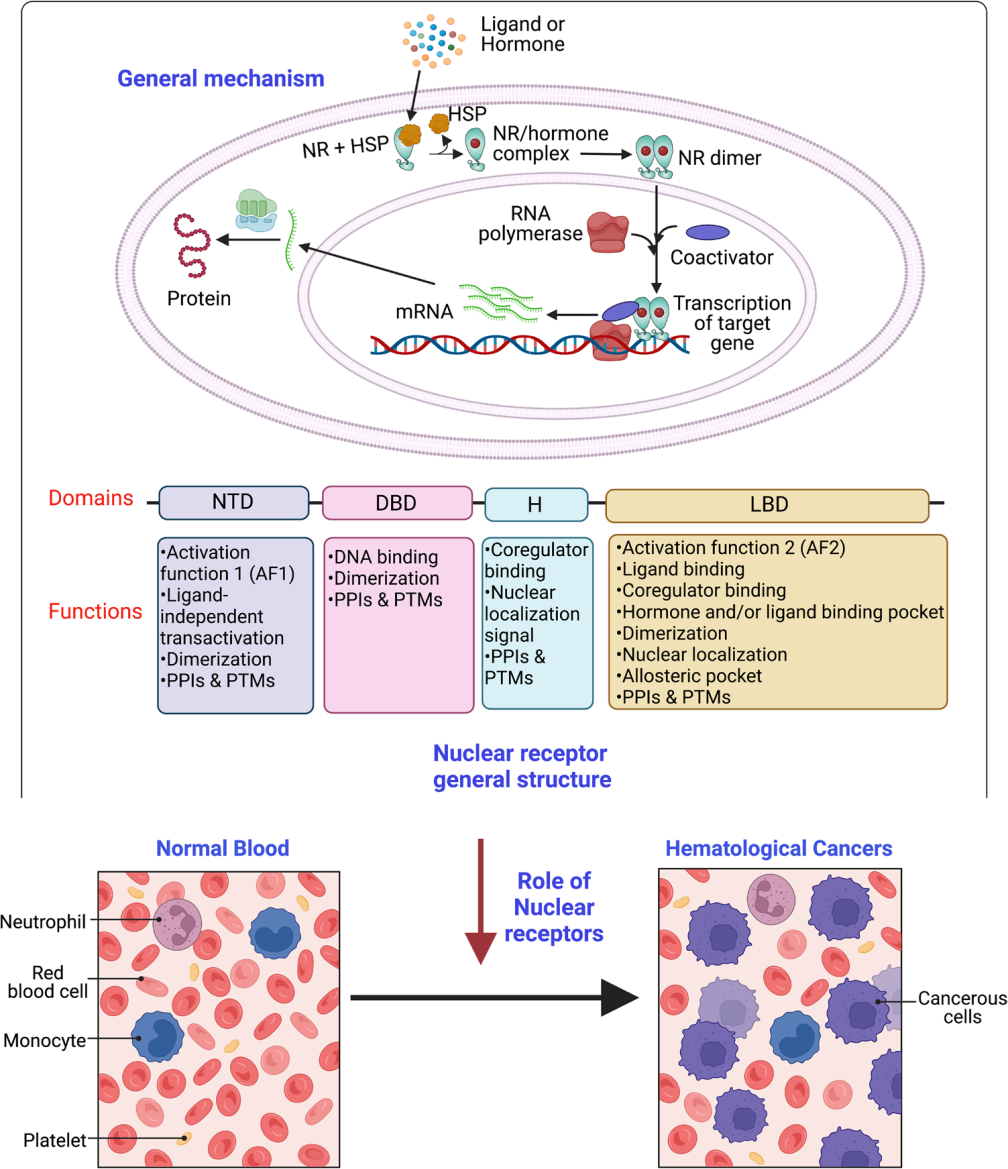

## Introduction

Hematological malignancies (HM) comprise a spectrum of malignancies that originate in the blood, bone marrow, and lymphatic systems. Historically, HM has been a focal point in oncological research, pioneering the incorporation of genetic evaluations to assist in diagnosis, categorization, prognosis and therapeutic selection. Genetic characterization plays a pivotal role in the clinical assessment of almost all types of HM, and it has consistently evolved alongside the advancements in molecular diagnostic technologies and cancer genomics [1]. Leukemia, myeloma, lymphoma, myelodysplastic syndromes, and myeloproliferative neoplasms represent the principal classifications of HM. Further, there exists a diverse array of subtypes within both the lymphoma and leukemia categories, as well as numerous less prevalent hematological tumors, each with distinct categorizations [2]. Leukemia, one of the main subtypes of HM, predominantly consisting of chronic myeloid neoplasm and acute leukemias. Lymphoma, another type of HM that originates in the lymphatic system is categorized into two main subtypes including hodgkin lymphoma and non-hodgkin lymphoma. Further hodgkin lymphoma is divided into classical and non-classical types and non-hodgkin lymphoma is  divided into B cell, T cell, and natural killer (NK) cell types [3]. Multiple myeloma (MM) arises from the uncontrolled proliferation of plasma cells within the bone marrow resulting in the overproduction of aberrant dysfunctional immunoglobulins which ultimately leads to anemia, bone lesions, hypercalcemia, etc. [4]. Recent research has found that HM exhibit recurrent methylation-related mutations, anomalous DNA methylation profiles, and aberrant histone deacetylase expression with a particular emphasis on their prevalence in leukemia and lymphoma. The alteration of DNA methylation profiles is commonly observed in two phases, methylation level and mutations of methyltransferase genes or demethylase genes [5, 6]. These alterations in epigenetic modifiers lead to positive feedback and a glide away from the tight regulation of epigenetic setpoint, that causes remodeling in a niche, autoregulating cell growth and survival [7, 8]. Similarly, histone modifications such as acetylation and methylation are most importantly involved in chromatin state regulation. Anomalous histone deacetylation is one of the many factors responsible for disrupted gene silencing. In leukemogenesis, aberrant recruitment of histone deacetylases 1/3 (HDAC 1/3) by AML1-ETO and PML-RARα fusion genes leads to the repression of genes involved in the differentiation of hematopoietic cells [8–10]. Lymphoma and leukemia, are presently subjected to a wide array of therapeutics, either as monotherapies or in combination regimens. These approaches include chemotherapy, chimeric antigen receptor-T cells (CAR-T cells), immunotherapy, targeted therapies, immune checkpoint modulators, and phytochemicals etc., in both clinical and pre-clinical settings [11–33]. Additionally, hormonal therapy also tend to show anticancer properties like apoptosis, antiinflammatory, antifibrosis, etc. [34]. Through the application of these therapeutic agents to patients with HM, there has been a significant enhancement in patient outcomes, with some cases witnessing complete remission in conditions such as CML and promyelocytic leukemia [11]. However, certain subtypes of leukemias and lymphomas persist as substantial public health challenges due to their resistance to modern treatments, which is a significant factor in HM recurrence and treatment failure [11]. Irrespective of the therapeutic modality, malignant hematopoietic cells frequently undergo genomic and intracellular adaptations to circumvent the effects of therapeutic agents [11]. Furthermore, the signaling pathways that controls apoptosis, autophagy, cellular differentiation, proliferation, epigenetic modifications, and the interplay of tumor suppressor genes and oncogenes might contribute to the emergence of therapy-induced resistance [11]. Mechanisms like cytokine and growth factor production or exosomal secretion, in the tumor microenvironment (TME) might also be the reason for resistance mechanisms in HM [11]**.** GLOBOCAN 2020 estimated 474,519 new cases and 311,594 deaths worldwide in leukemia and 176,404 new cases and 117,077 deaths in multiple myeloma [35]. Consequently, there is a pressing need to develop novel therapeutic strategies that can subdue the hurdles of resistance of cancer cells and toxicity of the existing medications.

Historically, nuclear receptors (NRs) have been playing a crucial role in oncogenesis, acting as important regulators in the disease process [36]**.** The NR superfamily encompasses a broad group of transcription factors (TF) with 48 members and is categorized into six distinct subfamilies, based on their evolutionarily conserved sequences and structures [37, 38]. The first subfamily encompasses thyroid hormone receptor-like members, including vitamin D receptor (VDR), thyroid hormone receptor (THR), retinoic acid receptors (RARs), all peroxisome proliferator-activated receptors (PPARs), and orphan receptors like RORs, Rev-Erb receptor, pregnane X receptor (PXR), liver X receptor (LXR), constitutive androstane receptor (CAR), and others. The second group comprises the retinoid X receptor (RXRs) and hepatocyte nuclear factor 4 (HNF-4). The third subfamily houses estrogen receptor-like members like the androgen receptor (AR), glucocorticoid receptor (GR), mineralocorticoid receptor (MR), progesterone receptor (PR), and the estrogen-related receptor (ERR), essentially encompassing the sex and adrenal steroid receptors. The fourth category integrates members including NGFI-B, Nor1, and Nerr1. The fifth subfamily is more concise, constituting the steroidogenic factor-like receptors, NR5A1 and NR5A2. Finally, the sixth category consists solely of receptors not aligned with the preceding subfamilies, designated as the germ cell nuclear factor 1 (GCNF1) [39]. The NRs basic structure comprises an N-terminal regulatory domain (NTD) featuring the activation function (AF1), a ligand-binding domain (LBD), a DNA-binding domain (DBD), and an intervening hinge region [37, 40]. Certain NRs recognize and bind to conserved DNA sequences named hormone response elements (HRE) either as monomers, homodimers, or heterodimers. These receptors can also be categorized into four distinct types based on their mode of action. The type I receptors including AR, ER, and PR, are sequestered in the cytoplasm by chaperone proteins and are released upon ligand binding to form homodimers, that expose nuclear localization signal (NLS) facilitating nuclear translocation and activation of its target genes through interaction with transcriptional coactivators [41–44]. Type II receptors such as THR and RAR lie within the nucleus in the absence of its ligand, bound to HREs repressing the transcription of its target genes through interacting with SMRT and NCoR corepressor complexes. It gets activated in the presence of its ligand leading to the activation of its target genes through interacting with coactivator complexes containing histone acetyltransferases replacing corepressor complexes [42, 45–47]. Type III receptors function similarly to type I receptors, where the former binds to direct repeats and the latter binds to inverted repeats [48]. Type IV receptors bind to HREs as monomers [48]. Cofactors that facilitate NR signaling through epigenetic alterations determine the target gene expression thereby regulating various cellular processes.

**Fig. 1:**
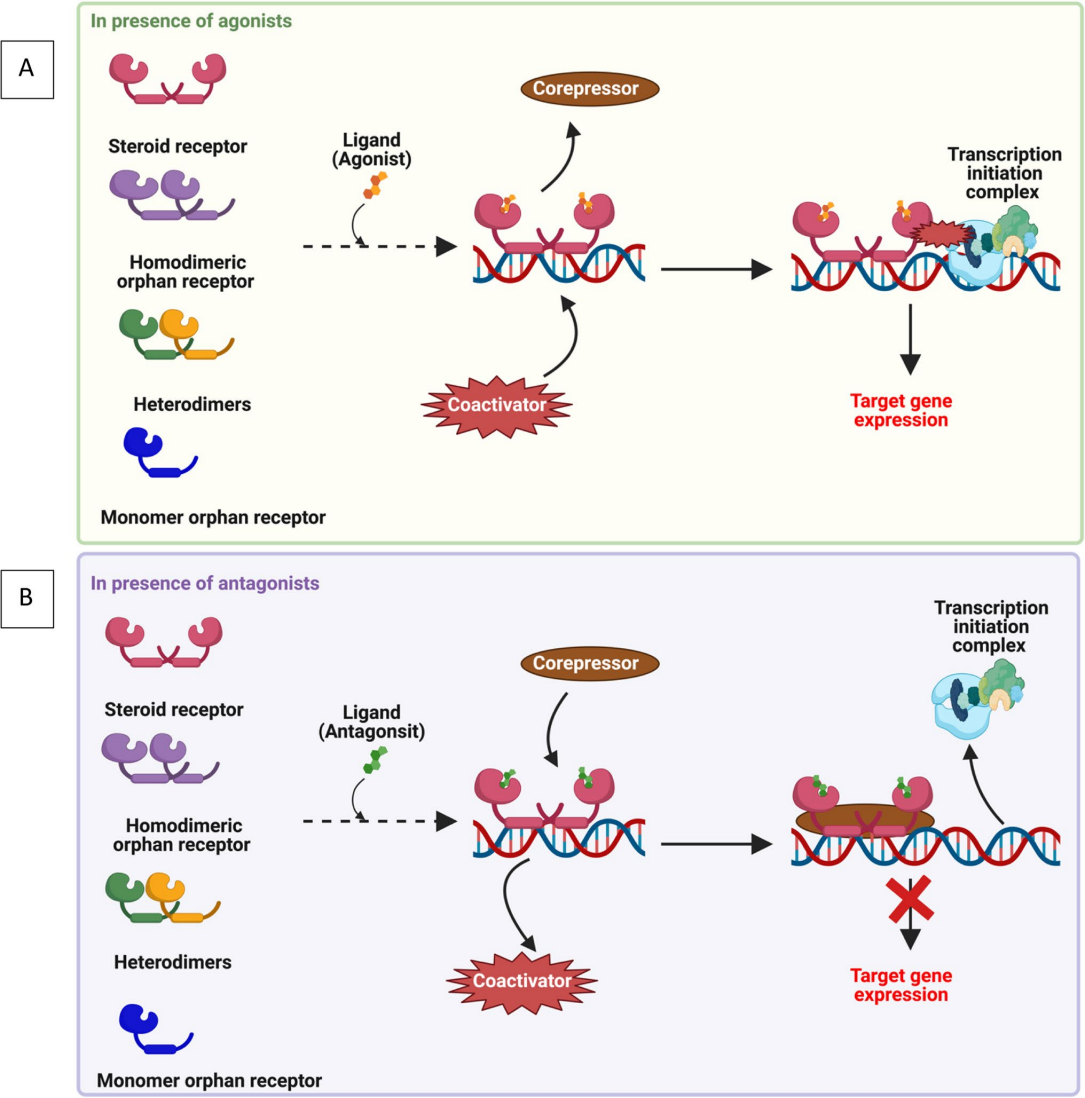
Nuclear receptor general signaling mechanism: **a** In the presence of agonists; **b** In the presence of antagonists. The ligands affiliated with nuclear receptors can exhibit either agonistic or antagonistic properties. Agonists, upon binding, induce structural alterations in nuclear receptors, facilitating the association with coactivators. This interaction subsequently triggers the transcriptional activation of downstream target genes. In contrast, the binding of antagonists hinders the transcription of target genes by engaging corepressors, leading to transcriptional suppression

NRs play an essential role in controlling cellular differentiation, embryogenesis, homeostatic maintenance, and the regulation of numerous downstream signaling pathways and physiological events **(**Fig. 1**)** [38, 49]. An accumulating number of studies have proved the significant role of NRs in the management of different hallmarks of cancers [38, 50, 51]. FDA-approved drugs, like toremifene, raloxifene, and bicalutamide, are designed to target NRs for the treatment of different malignancies [52–55]. Therefore, in this review, we highlight the significance of NRs in HM, emphasizing their interaction with a variety of agonists, antagonists, and other selective modulators. We further explore their influence on tumor-related processes such as proliferation, differentiation, invasiveness, migration, apoptosis, etc.

## NRs in HM

NRs are traditionally characterized as TFs activated by specific ligands, playing crucial roles in reproduction, development, and various physiological processes. In humans, there exist 48 such receptors, and their dysregulation frequently correlates with pathological conditions. Given that the majority of these receptors can be selectively modulated by small molecular entities, they stand as significant targets for therapeutic interventions [56]. According to the literature, several PPAR agonists, vitamin D derivatives, and all-trans retinoic acid (ATRA) have been identified to modulate apoptosis, differentiation, and cell proliferation in distinct leukemic cells [37]. When these compounds are introduced either alone or in combination, showed improved outcomes, thereby suggesting the participation of multiple NRs in the pathophysiology of leukemia. Additionally, numerous NR agonists, encompassing vitamin D and PPAR activators, have the potential to enhance the efficacy of existing therapeutic regimens [37]. The primary reason for employing NRs in therapeutic approaches is the limitation of traditional methods, like chemotherapy. Conventional regimens primarily target cancer cells but concurrently affect healthy cells, including bone marrow stem cells, gastrointestinal epithelial cells, and progenitor cells, causing significant toxicity in patients [37].

Specific NRs are of great interest as therapeutic targets in myeloid leukemia due to their involvement in modulating myeloid cell differentiation. The profound effectiveness of ATRA in managing APL serves as an ideal and unmatched model of cancer differentiation therapy in advanced research [37]. ATRA acts on RAR to promote apoptosis and differentiation of leukemic cells [37]. In addition, ligands specific to VDR and PPAR stimulate apoptosis and curtail leukemic cell proliferation. Notably, the combined use of NR agonists/antagonists with chemotherapeutic agents has been observed to augment antileukemic activities [37, 57, 58].

Certain NRs show cancer-specific behaviors, meaning their roles and expression patterns vary across cancer types. For instance, the ER shows elevated expression in oral cancer, while its expression varies in lung cancer [50, 51]. Similarly, VDR exhibits diminished expression in HM but presents differential expression in esophageal cancer [38, 59, 60]. Additionally, distinct isotypes of specific NRs, like PPAR, have unique functional implications. In the context of PPAR, Cheng et al. proposed an oncogenic role for PPARα [61]. A focused cross-sectional analysis of 100 patients revealed that increased PPARα expression in the colorectal cancer  TME was indicative of an adverse prognosis [62]. On the contrary, multiple studies have highlighted a potential anticarcinogenic role for PPARγ [63, 99]. Yaghoubizadeh et al. suggested that, enhanced PPARγ expression is linked to favorable outcomes in colorectal cancer patients [62]. However, a thorough analysis of the functions and implications of each NR in diverse cancers is crucial for the development of efficient therapeutic strategies.

**Fig. 2:**
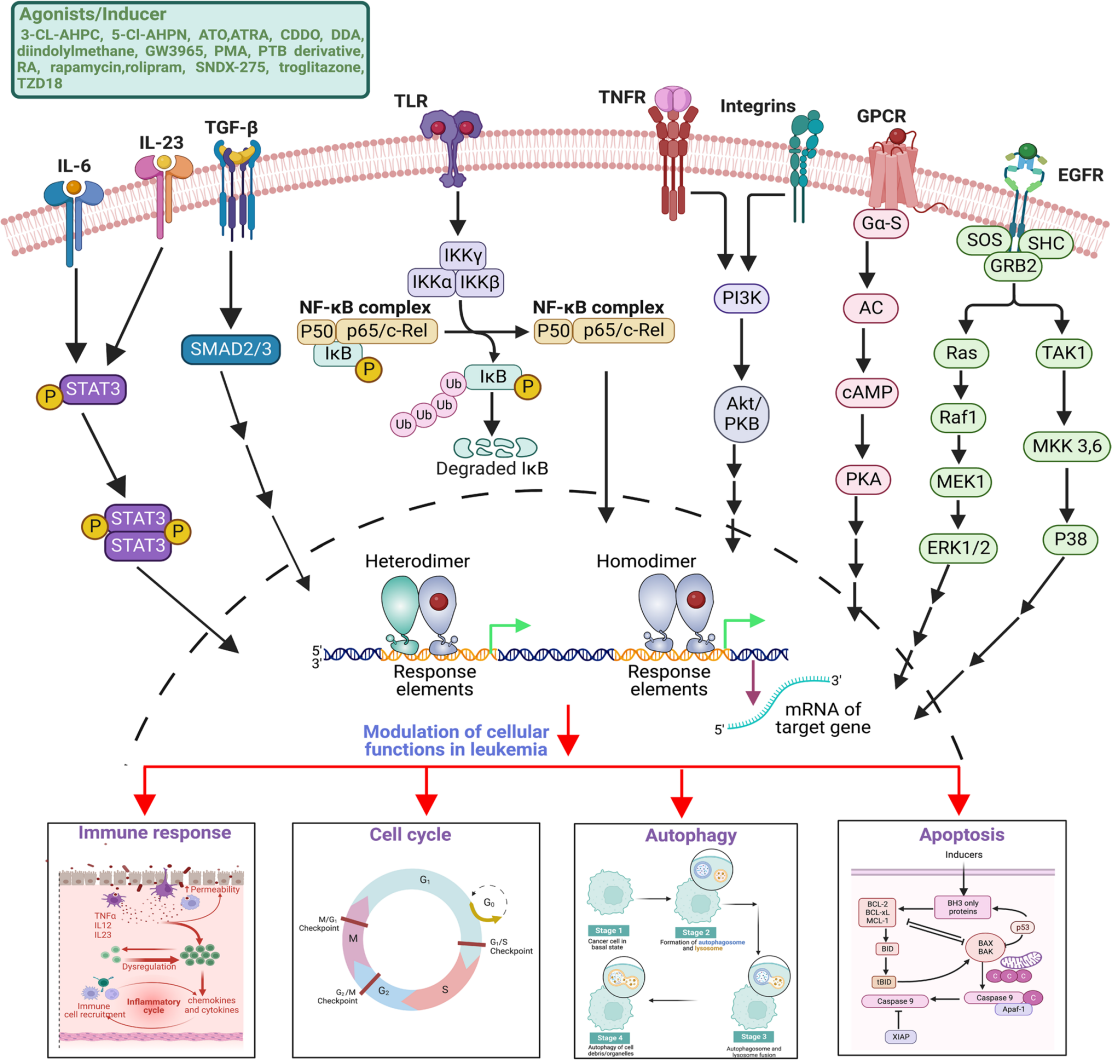
Nuclear receptors and their ligands that are involved in the modulation of cellular functions in leukemia. Numerous investigations have elucidated the pivotal role of nuclear receptors in the modulation of cellular signaling processes. The exploration of nuclear receptor functions in the context of leukemic cell characteristics has been conducted employing diverse inducers or agonists. Through these mechanistic inquiries, the engagement of key molecular entities such as TNF, TGF-β, EGFR, integrins, STAT3, and TLR has been unveiled, shedding light on their intricate involvement in the initiation and advancement of leukemia

**Fig. 3:**
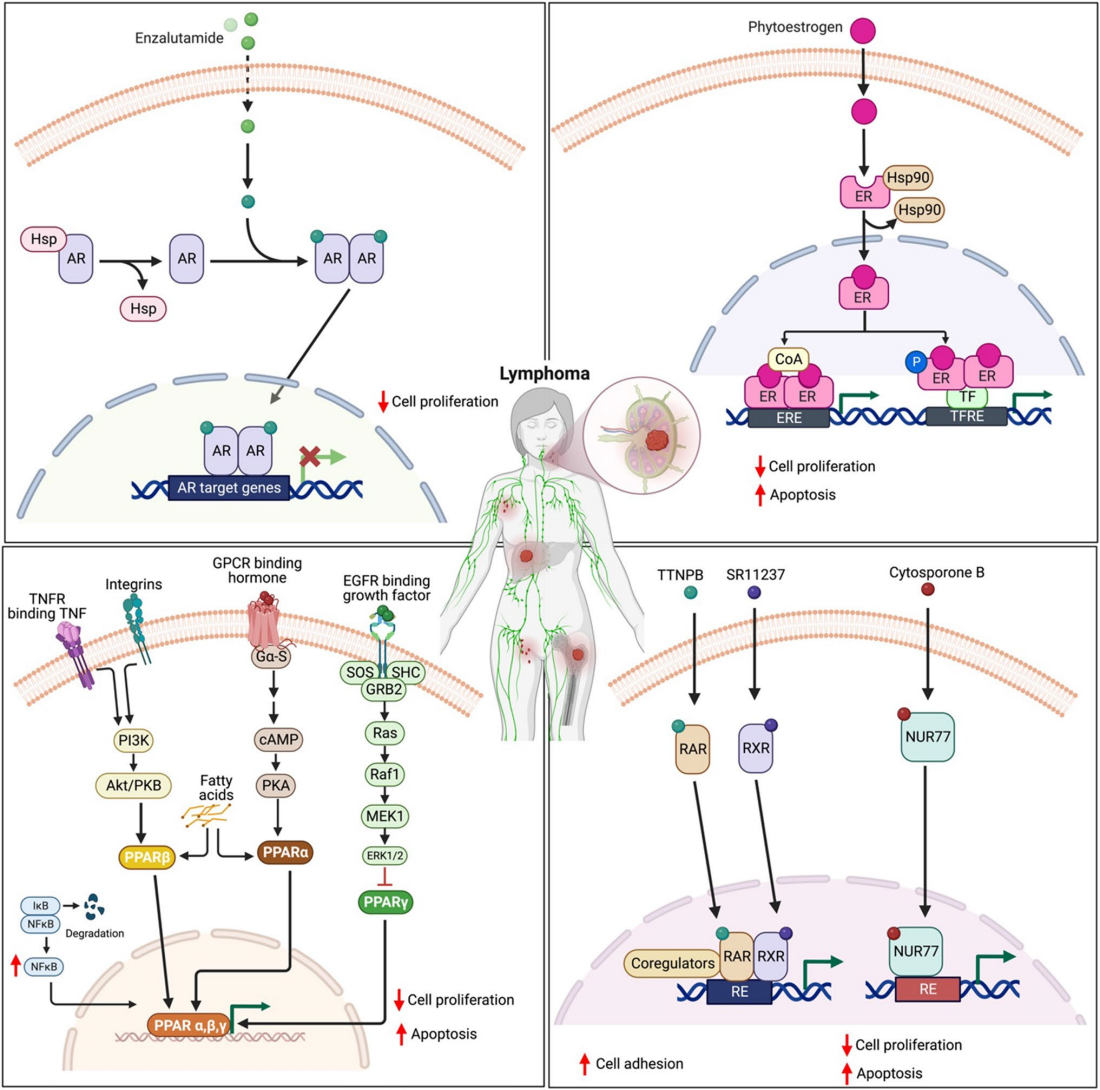
Nuclear receptors and their ligands that are involved in the modulation of cellular functions in lymphoma. Scientific investigations have provided compelling evidence demonstrating the participation of nuclear receptors, specifically AR, ER, PPARα, PPARβ, and PPARγ, in the regulation of key characteristics of lymphoma cells. Through functional studies based on ligand interactions, it has been established that these nuclear receptors play a pivotal role in modulating the proliferation and apoptosis of lymphoma cells

**Fig. 4:**
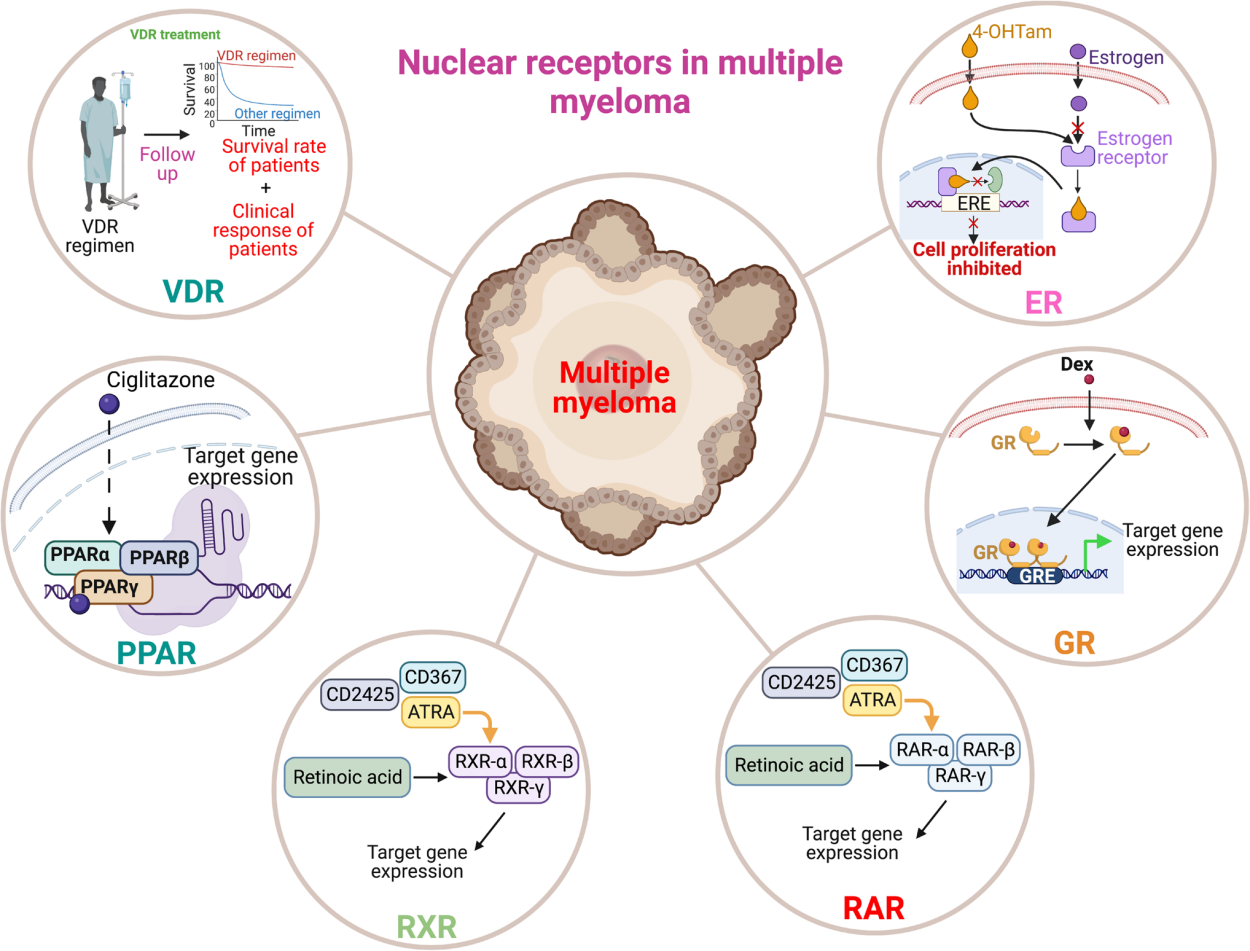
Nuclear receptors and their ligands that are involved in the modulation of cellular functions in multiple myeloma. Nuclear receptors, including ER, GR, RAR, RXR, PPAR, and VDR, have been demonstrated to exert significant influence over myeloma cell growth, proliferation, and apoptosis. Notably, the administration of a VDR agonist, specifically dexamethasone, has been proven to augment both the overall response rate and progression-free survival in affected patients. Consequently, these receptors emerge as promising therapeutic targets for the treatment of multiple myeloma

Moreover, there exists a notable gap in the comprehensive literature that encapsulates the significance of NRs in therapeutic strategies for HM. This review focusses on how  altered HM cells can be targeted through the use of agonists and antagonists, emphasizing the potential of NRs as a novel paradigm in both the treatment and management of HM. The NRs implicated in HM include AR, ER, GR, LXR, Nur77, Nor1, PPAR, RAR, RXR, SHP, and VDR. This review extensively highlights the expression profile (Table 1) of these NRs along with the mechanistic interaction regulating various cancer hallmarks (Figs. 2, 3, 4) (Tables 2, 3, 4).

**Table 1:** Studies on the expression analysis of nuclear receptors in hematological malignancy

Nuclear receptors (NRs)	In vitro/In vivo/In silico/Clinical	Models/cell lines/tissue/condition	Expression (down-/up-regulation)	References
ERβ2	Clinical	CLL B cell	Up	[64]
ERRα	Clinical	AML patients	Up	[65]
GRα	Clinical	MM patients	Up	[66]
RXR	Clinical	Leukemia	Down	[60]
	Clinical	Lymphoma	Down	[60]
VDR	Clinical	Leukemia	Down	[60]
	Clinical	Lymphoma	Down	[60]
	Clinical	ALL	Down	[59]

**Table 2:** Mechanistic role of various nuclear receptors in Leukemia in the presence of their agonists/antagonists (^A^Agonist, ^B^Antagonist, ^C^Inverse agonist)

Nuclear receptors (NRs)	In vitro*/*In vivo*/ *In silico*/*Clinical	Models/cell lines/condition	Agonist/Antagonist	Results	References
ER	Clinical	CLL patients	Busramustine^A^ (KM-2210)	↑ Survival rate ↓ Number of enlarged lymph nodes, peripheral blood lymphocytes	[67]
ERRα	In vitro	KG-1α cells	XCT-790^C^	↑ Apoptosis, cleaved Caspase-9 ↓ Cell viability, ERRα, NDUFS3, UQCRFS1, COX5A, COX5B	[65]
	In vivo	NOD/SCID mice subcutaneously injected with KG1α cells	shERRα-KG1α	↓ Tumor volume	[65]
	In vivo	NIG mice injected with shERRα-KG1α	–	↑ Survival rate	[65]
GR	In vitro	CEM cells	small molecule 2-(4-acetoxyphenyl)-2-chloro-N-methylethylammonium-chloride^B^ and Bortezomib	↓ Cell growth, NF-κB, AP-1	[68]
	Clinical	Myeloid leukemia, ALL, CLL Patients	Dex^A^	↓ GR sites	[69]
	In vivo	T lineage acute lymphoblastic leukemias (T-ALLs) xenografts	Dex^A^ , Dex^A^ + GDC0941	↑ Survival	[70]
	In vitro	Jurkat, Nalm-6 cells	Huai Qi Huang (HQH)	↑ Apoptosis, cleaved Caspase-3, Bax, GRα ↓ Cell survival, Bcl-2, pERK	[71]
	In vitro	Jurkat, Nalm-6 cells	HQH + Dex^A^, HQH+PD98059	↑ Apoptosis, Bax, cleaved Caspase-3, GILZ, NFKBIA, GRα ↓ Bcl-2, pERK	[71]
	In vitro	CEM-C1 cells	Rapamycin	↑ Cell cycle arrest G0/G1 phase ↓ Cell growth	[72]
	In vitro	CEM-C1 cells	Rapamycin + Dex^A^	↑ Apoptosis, Cell cycle arrest G0/G1 phase, GRα, p-GR, Bim-EL ↓ Cell Growth, p-p70S6K, mTOR	[72]
	In vitro	B-CLL cells	Rolipram, Cilomilast, Roflumilast	↑ GRα, Apoptosis	[73]
	In vitro	B-CLL cells	Actinomycin-D + Rolipram	↑ GRα	[73]
	In vitro	B-CLL cells	Rolipram + Dex^A^	↑ Apoptosis, Relative GRα transcript	[73]
LXR	In vitro	KG-1, HL-60 cells	DDA^A^	↑ Autophagy	[74]
	In vivo	NSG mice (Primary AML cell xenografts)	DDA^A^	↑ Nur77, Nor1 ↓ Leukemic burden, Number of AML cells, Cell viability in both bone marrow and spleen	[74]
	In vitro	KG-1, KG-1a, MV4-11 cells	Daunorubicin + DDA^A^	↑ Cell death	[75]
	In vitro	KG1a, OCI-AML3, MOLM14 cells	Idarubicin + DDA^A^	↑ Cell death, Autophagy	[75]
Nor1	In vitro	HL-60, MOLM13, OCI-AML3, OCI-AML2 cells	SNDX-275	↑ TRAIL, Apoptosis, Bim, Noxa, c-Jun, JunB, Nor1	[76]
	In vitro	HL-60, Kasumi-1 cells	Z-LIG	↑ Apoptosis, Nor1 ↓ Proliferation, Colony formation	[77]
	In vivo	NOD/SCID mice (Silencing of Nur77 and Nor1)	Z-LIG	↓ Anti-AML activity of Z-LIG	[77]
Nur77	In vitro	HL-60 cells	Cantharidin	↑ Apoptosis, cleaved Caspase-3, -8, PARP, Cell cycle arrest at G2/M phase, Differentiation, Nur77 ↓ Cell viability, Colony formation, Proliferation	[78]
	In vivo	NOD/SCID mice (HL-60) xenograft	Cantharidin	↑ Survival, RBC count ↓ Liver weight, Spleen weight, WBC count	[78]
	In vitro	HL-60, MOLM13, OCI-AML3, OCI-AML2 cells	SNDX-275	↑ TRAIL, Apoptosis, Bim, Noxa, c-Jun, JunB, Nur77	[76]
	In vitro	HL-60 cell	Azacytidine + Metacept-1	↑ Apoptosis, p15^INK4b^, p21^WAF1/CIP1^, Caspase-3, Nur77 ↓ MMP-9, Cell growth, Bcl-xl	[79]
	In vitro	HL-60, Kasumi-1 cells	Z-LIG	↑ Apoptosis, Differentiation, Nur77 ↓ Proliferation, Colony formation	[77]
	In vivo	NOD/SCID mice (Silencing of Nur77 and Nor1)	Z-LIG	↓ Anti-AML activity of Z-LIG	[77]
	In vitro	HL-60 cells	Ginsenoside 20(S)-Rh2	↑ Apoptosis, Differentiation, Nur77	[80]
	In vivo	NOD/SCID mice (HL-60 cells xenografts)	Ginsenoside 20(S)-Rh2	↑ Differentiation, Nur77 ↓ Splenomegaly	[80]
PPAR	In vitro	HL-60 cells	Troglitazone^A^	↑ Differentiation to monocytes, G1 arrest ↓ Cell growth,	[81]
	In vitro	HL-60 cells	Troglitazone^A^	pRb ↑ Apoptosis, cleaved Caspase-3 ↓Cell proliferation, c-myc, Tcf4	[82]
	In vitro	HL-60, K562 cells	15d-PGJ2^A^, Troglitazone^A^	↑ Apoptosis, Bax ↓ Survivin, Bcl-2	[83]
	In vitro	Mono Mac6, U937 cells	15d-PGJ2^A^, Troglitazone^A^	↑ Apoptosis, Caspase-3, cleaved PARP, Bax ↓ Mitochondrial membrane potential, COX-2, Bcl-2, Bcl-xl, Mcl-1	[84]
	In vitro	U937 cells	1,1-bis[3′-(5-methoxyindolyl)]-1-(p-t-butylphenyl) methane^B^ (DIM #34^B^)	↑ Apoptosis, cleaved Caspase -3, -8 , -9, p18 Bax ↓ Cell growth, Mitochondrial membrane potential, p-Bcl-2, p-ERK, MBP-PO_4_	[85]
	In vitro	Jurkat cells	DIM #34^B^	↑ cleaved Caspase-3, -9	[85]
	In vitro	HL-60 cells	DIM #34^B^	↑ Apoptosis	[85]
	In vitro	U937 cells	DIM#34^B^ + LG100268, DIM#34^B^ + LG100069, DIM#34^B^ + ATRA	↑ Apoptosis	[85]
	In vitro	Jurkat cells	15d-PGJ2^A^	↑ DR5, TRAIL induced apoptosis	[86]
	In vitro	EoL-1, U937, KPB-M15 cells	Troglitazone^A^	↑ p21 ↓ Cell proliferation	[87]
	In vitro	EoL-1 cells	Troglitazone^A^	↑ Cells arrest, G0/G1 phase population	[87]
	In vitro	CLL cells, Jurkat cells	CDDO^A^	↑ Apoptosis	[88]
	In vitro	HL-60 cells	Troglitazone^A^	↑ Apoptosis, Monocytic differentiation, G0/G1 arrest, Bax ↓ Cell proliferation, Bcl-2	[89]
	In vitro	HL-60 cells	Troglitazone^A^ + LG100268 , Pioglitazone^A^ + LG100268	↓ Cell proliferation	[89]
	In vitro	HL-60 cells	Troglitazone^A^ + LG100268	↑ Bcl-xl, CD11b, CD14, Bax ↓ Bcl-2	[89]
	In vitro	HL-60, U937, Su-DHL, Sup-M2, Ramos, Raji, HD-MYZ, HD-LM-2, L-428, KM-H2, and primary chronic lymphocytic leukemia cells	Rosiglitazone^A^, 15d-PGJ2^A^, CDDO^A^	↓ Cell Viability	[90]
	In vitro	U937 cells	CDDO^A^	↑ Apoptosis, cleaved Caspase-3, -8, -9	[90]
	In vitro	THP-1, U937 cells	Troglitazone^A^ + ATRA	↑ Differentiation, Phagocytic ability	[90]
	In vitro	HL-60, NB4, U937, THP-1, ML-1 cells	Troglitazone^A^	↓ Clonal growth	[91]
	In vitro	U937 cells	Troglitazone^A^	↑ Cells in G1 phase cell cycle ↓ Clonal growth	[91]
	In vitro	HL-60, NB4, U937, THP-1, ML-1 cells	Troglitazone^A^ + 9-cis RA	↓ Clonal growth	[91]
	In vitro	HL-60 cells	CDDO-Me^A^	↑ Apoptosis, Bax	[92]
	In vitro	U937 cells	CDDO-Me^A^	↑ Bax, Apoptosis, Induced decrease in the mitochondrial membrane potential, ↓ p-ERK1/2	[92]
	In vitro	HL-60, KG-1, NB4 cells	CDDO-Me^A^ + ATRA	↓ Viable cells	[92]
	In vitro	HL-60 cells	CDDO-Me^A^ + LG100268	↑ Apoptosis	[92]
PPAR	In vitro	U937, HL-60 cells	CDDO^A^	↑ Apoptosis, Caspase-8	[93]
	In vitro	U937 cells	CDDO^A^ and ara-C	↑ Cyt c, cleaved Caspase-3, PKCδ	[93]
	In vitro	Primary CLL B cells	CDDO^A^	↑ Apoptosis, cleaved Caspase-3, -8, -9 ↓ FLIP	[94]
PPAR	In vitro	MJ, Hut78, and HH cells	CDDO^A^	↓ Cell growth, ↑ Apoptosis, sub-G1 population	[95]
	In vitro	Jurkat, J-Jahn cells	15d-PGJ2^A^, PGD2	↑ Apoptosis ↓ Proliferation	[96]
	In vitro	HL-60 cells	CDDO^A^	↑ Apoptosis, Granulocytic-monocytic differentiation, Phagocytosis, CD11b, CD11c, CD16, p21^cip1/waf1^, G-CSFR, CEBPA DNA binding, p42/p30 CEBPA ↓ c-myc	[97]
	In vitro	U937 cells	CDDO^A^, CDDO-Me^A^, CDDO-Im^A^	↑ Mitochondrial Dysfunction, Cyt c, Apoptosis, JNK, Intracellular ROS levels, cleaved Caspase-8 ↓ Intracellular GSH levels,	[98]
	In vitro	UTree-O2, Bay91, 380 cells	Troglitazone^A^	↑ Apoptosis, G1 arrest ↓ Cell growth, c-myc expression	[99]
	In vitro	HL-60 cells	CDDO^A^	↑ Apoptosis, Differentiation, Cell cycle arrest	[100]
	In vitro	U937 cells	CDDO^A^	↑ cleaved Caspase-3, -8, -9, Apoptosis ↓ Mitochondria membrane potential	[100]
	In vitro	Jurkat cells	CDDO^A^	↑ Apoptosis	[100]
	In vitro	HL-60, MO7e, NB4, U937, OCI-AML3, KBM3 cells	CDDO^A^	↓ Cell number	[100]
PPAR	In vitro	K562 cells	Troglitazone^A^	↓ Cell number, GATA-1, Glycophorin A	[101]
	In vitro	K562 cells	Troglitazone^A^ + LG100268	↓ Cell number, GATA-1, Glycophorin A	[101]
PPAR	In vitro	KU812, KCL22 cells	TZD18 + Imatinib	↑ Apoptosis ↓ Cell growth	[102]
	In vitro	K562, KCL22, KU812 cells	TZD18^A^	↓ Proliferation	[102]
	In vitro	KUB12 cells	TZD18^A^	↑ Bax, p27 ↓ Cyclin D2, Cyclin E, CDK2	[102]
PPAR	In vitro	NB4, MR2 cells	CDDO^A^+ ATRA	↑ Apoptosis, Differentiation ↓ Viable cells	[103]
	In vitro	NB4, MR2 cells	CDDO^A^	↑ Apoptosis ↓ Viable cells	[103]
PPAR	In vitro	THP-1 cells	Troglitazone^A^, Rosiglitazone^A^	↓ MCP-1-directed THP-1 monocyte migration, MMP-9, MCP-1-directed THP-1 monocyte chemotaxis	[104]
PPAR	In vitro	THP-1 cells	9-cis RA	↑ PPAR*γ*1 RNA, Nuclear PPAR*γ*1 protein ↓Cell growth	[105]
	In vitro	THP-1 cells	9-cis RA + BRL49653^A^	↑ G1 phase cell population ↓Cell growth	[105]
	Clinical	AML patients blasts	CDDO^A^	↑ Ratio of p42/p30 CEBPA in a subset of patients	[97]
	In vitro	Chronic phase CML CD34^+^ cells	Imatinib + Pioglitazone^A^	↓ STAT5, HIF2α, CITED2	[106]
	Clinical	CML patient samples	Pioglitazone^A^	↓ Clonogenic potential of bone marrow CD34^+^ cells, STAT5	[106]
RARα	In vitro	HL-60, NB4 cells	PTB^A^	RXR*α*/RXR*α* and partially activated PPAR*γ*/RXR*α,*RAR*α*/RXR*α,*PPARδ(β)/RXR*α* ↑ Differentiation ↓ Cell growth, Proliferation	[107]
	In vitro	NB4 cells	ATRA, 9-cis RA	↑ Maturation, CD11c	[108]
	Clinical	APL patients	ATRA^A^ + ATO	↑ Complete remission, Event-free survival, Disease-free survival ↓ Relapse probability	[109]
RAR	In vitro	HL-60 (RA resistant RAR alpha overexpressing) cells	ATRA^A^	↑ Differentiation, Apoptosis	[110]
	Clinical	AML patients	ATRA^A^ + APO + Idarubicin	↑ Relapse-free & failure-free survival	[111]
	Clinical	APL patients	ATRA^A^ + ATO	↑ Complete remission, Event-free survival rates, Overall survival rates	[112]
	In vitro	K562 cells	DAC + ATRA^A^	↑RAR-β, p16 demethylation	[113]
RAR	Clinical	APL	ATRA^A^ + ATO	↑ Complete remission, disease-free survival	[114]
	In vitro	NB4 cells	BMS753	↑ Maturation	[108]
RXR	Clinical	AML	Bexarotene^A^	↓ Bone marrow blasts, ↑ Platelet counts, Neutrophil count	[115]
	In vitro	HL-60 (RA resistant RXR alpha overexpressing) cells	9-cis RA^A^	↑ Differentiation, Apoptosis	[110]
	In vitro	HL-60 (blr1 overexpressing) cells	RA^A^	↑ p-ERK2, Myeloid differentiation, G1/G0 arrest	[116]
	In vitro	HL-60, KG-1, THP-1, WEHI-3 cells	TTAB + SR11217^A^	↓ Clonal growth	[117]
	In vitro	HL-60 cells	TTAB	↑ Differentiation, CD11b	[117]
	In vitro	HL-60 cells	SR11236, SR11246^A^	↑ Differentiation ↓ Clonal growth	[118]
	In vitro	HL-60 cells	SR11249, SR11256^A^, LGD1069^A^	↑ RXR/RXR homodimers and RAR/RXR heterodimers, Differentiation ↓ Clonal growth	[118]
RXRα	In vitro	HL-60 cells	ATRA^A^ + (SR11249 or SR11256^A^, or LGD1069^A^)	↑ Differentiation	[118]
	In vitro	HL-60, NB4 cells	PTB^A^	RXR*α*/RXR*α* and partially activated PPAR*γ*/RXR*α,*RAR*α*/RXR*α,*PPARδ(β)/RXR*α* ↑ Differentiation ↓ Cell growth, Proliferation	[107]
	In vitro	HL-60 cells	13-cis RA^A^, ATRA^A^, 9-cis RA^A^	↑ Differentiation, CD11b, ↓ Proliferation	[119]
	In vitro	HL-60 cells	ATRA^A^, 9-cis RA^A^	↓ c-myc	[119]
	In vitro	HL-60 cells	9-cis RA^A^	↓ RXRα	[119]
	In vitro	ML-1, HL-60, NB4, THP-1, U937, KU812 cells	Differanisole A	↓ Cell growth	[120]
	In vitro	HL-60 cells	Differanisole A + (VD3 or ATRA^A^ or 9-cisRA)	↑ Differentiation, CD11b ↓ Cell growth, NBT +ve cells	[120]
	In vitro	HL-60 cells	Differanisole A + (AM80 or Ro47-5944^A^)	↓ Cell growth	[120]
	In vitro	HL-60 cells	9-cis RA^A^, ATRA^A^	↑ Differentiation ↓ Cell growth	[121]
	In vitro	NB4 cells	9-cis RA^A^, ATRA^A^	↓ Cell growth	[122]
	Clinical	APL Patients	9-cis RA (administered as LGD 1057)	Complete remission	[122]
RARα	In vitro	NB4 cells	Am 580^A^	↑ Cellular differentiation, growth arrest	[123]
RXR	In vitro	THP-1 cells	ATRA^A^	↑ MMP-2	[124]
	In vitro	THP-1 cells	ATRA^A^ + Dex	↓ ATRA-induced MMP-2 secretion	[124]
	In vitro	HL-60, NB4 cells	SR11278 (Retinoid D), SR11276^A^ (Retinoid E)	↓ Colony formation	[125]
	In vitro	HL-60 cells	ATRA^A^ + SR11278 (Retinoid D), ATRA^A^ + SR11276^A^ (Retinoid E)	↑ Differentiation	[125]
	In vitro	NB4 cells	ATRA^A^ + SR11276^A^ (Retinoid E)	↑ Differentiation	[125]
	In vitro	HL-60 cells	ATRA^A^	↑ CD11b	[125]
	In vitro	HL-60, NB4 cells	SR11278 (Retinoid D)	↑ RARα, RARβ, RARγ	[125]
	In vitro	HL-60, NB4 cells	SR11276^A^ (Retinoid E)	↑ RAR, RXR	[125]
SHP	In vitro	U937 cells	PMA	↑ p65, SHP, p21^WAF1^,c-Jun, p–c-Jun	[126]
	In vitro	U937 cells (SHP OE)	Etoposide	↓ Apoptosis, ↑ p21, Bax	[126]
	In vitro	U937 cells	Etoposide	↑ Apoptosis	[126]
	In vitro	KG-1 cells	3-Cl-AHPC, Compound40:Ac2O, DMAP, THF (1-Ad-substituted retinoid-related compounds)	↑ Apoptosis ↓ Cell growth	[127]
	In vitro	KG-1 cells	5-Cl-AHPN + 3-Cl-AHPC	↑ Apoptosis ↓ Cell growth	[128]
VDR	In vitro	U937 cells OE of Nrf 2	1,25D3^A^ , 1,25D3 + CA	↑ Transactivation of VDRE, VDR expression	[129]
	In vitro	MV-4–11, THP-1, HL-60 cells	Calcitriol^A^ , Tacalcitol^A^	↑ CD11b, CD14 ↓ Proliferation	[130]
	In vitro	HL-60, MOLM-13 cells	1,25D3 + Azacytidine, 25D3 + Azacytidine	↓ Cell proliferation	[131]
	In vitro	HL-60 cells	1,25D3, PRI-5202, 1,25D3+CA, PRI-5202 + CA, 1,25D3 + tBHQ, PRI-5202 + tBHQ, 1,25D3 + DMF, PRI-5202 + DMF	Synergistic effects, ↑VDR, Nrf2, CYP24A1, CAMP, TrxR1, CD11, CD14	[132]
	In vivo	SCID mice (HL-60 Xenografts)	PRI-5202 + DMF	↓ Tumour growth, Ki67 positive cells	[132]
	In vitro	HL-60 cells	1,25D3^A^ + SB415286	↑ Differentiation, CD11b, CD14 ↓ Colony formation, Cyclin A	[133]
	In vitro	OCI, THP-1, U937 cells	1,25D3^A^ + SB415286	↓ Colony formation	[133]
	In vitro	HL-60, cells (GSK3β knockdown cells)	1,25D3^A^	↑ Differentiation	[133]
	In vitro	OCI, HL-60 cells	1,25D3^A^ + SB415286	↑ CD14, p-MEK1/2, p-JNK, p–c-Jun	[133]

**Table 3:** Mechanistic role of various nuclear receptors in Lymphoma in the presence of their agonists/antagonists (^A^Agonist, ^B^Antagonist)

Nuclear receptors (NRs)	In vitro/In vivo/ In silico/Clinical	Models/cell lines/condition	Agonist/Antagonist	Results	References
AR	In vitro	Granta, Jeko-1, Rec-1, Maver-1 cells	Enzalutamide^B^	↓ MCL proliferation	[134]
ER *β*	In vitro	EGF7 (murine), Ramos, Raji cells	DPN^A^ or KB9520^A^	↓ Cell proliferation	[135]
	In vivo	C57Bl/6 J mice (EG7) xenograft	KB9520^A^ + ICI 182.780^B^	↓ Tumor volume	[135]
	In vivo	C57Bl/6 J mice (EG7) xenograft	DPN^A^ or KB9520^a^	↑ Apoptosis ↓ Tumor volume	[135]
	In vivo	NOD/SCID mice (Granta-519) xenograft	DPN^A^ or KB099520^A^	↓ Tumor growth, Ki67, BAFF, GRB7, VEGF-C, LYVE1, CD34, Angiogenesis, Lymphangiogenesis	[136]
	In vivo	Male NOD/SCID (Raji, Ramos cells) xenograft	DPN^A^	↓ Tumor growth	[136]
	In vivo	C57/BI6J mice (EG7 lymphoma T cell) xenograft	BPA^A^	↓ Tumor volume, Ki67	[137]
	In vivo	C57/BI6J mice (EG7 lymphoma T cell) xenograft	Genistein^A^	↑ Apoptosis ↓ Tumor volume, Ki67	[137]
	In vivo	NOD/SCID/IL2*γ* null mice (Granta-519 mantle cell) xenograft	BPA^A^ or Genistein^A^ or DPN^A^	↓ Tumor volume	[137]
GR	In vitro	NCEB, CEM cells	2-(4-acetoxyphenyl)-2-chloro-N-methylethylammonium-chloride^A^ + Bortezomib	↑ cleaved PARP ↓ Cell viability, S-phase cell %, NF-κB, AP-1	[68]
	Clinical	Non-Hodgkin’s lymphoma patients	Dex^A^	↓ GR sites	[69]
	In vitro	DoHH2 cells	Dex^A^	↑ Cell lysis	[138]
LXR	In vitro	CAL-1 cells	T0901317^A^, GW3965^A^	↑ Apoptosis, Bax, BAK1, LXRα, ABCG1, ABCA1, cleaved Caspase-3, Caspase-9 ↓ Cell proliferation, p-STAT5, p-Akt, Nuclear p50, p65, c-Rel	[139]
	In vivo	Sublethally irradiated (2 Gy) NSG mice were grafted with 1 million CAL-1 cells	T0901317^A^	↑ Mice survival ↓ Spleen size, CAL-1 infiltration in bone marrow and spleen	[139]
Nur77	In vitro	SuDHL4 cells	OE NR4A1	↑ NR4A1, Caspase-3, -7 activity, cleaved Caspase -3, Apoptosis, Sub-G1, Bim1/6, PUMA, TRAIL ↓ Cell growth	[140]
	In vitro	SuDHL4, Karpas422 cells	Cytosporone B^A^	↑ NR4A1, cleaved Caspase-3, Apoptosis, Sub-G1 ↓ Cell growth	[140]
	In vitro	U2932, RI-1, Karpas422 cells	OE NR4A1	↑ NR4A1, Apoptosis, Caspase-3, -7 activity, Sub-G1, Bim1/6, PUMA, TRAIL ↓ Cell growth	[140]
	In vivo	NSG mouse model (SuDHL4)	OE NR4A1	↓ Tumor growth	[140]
	In vitro	HH, MJ cells	Panobinostat	↑ Nur77, Nor1, BAK, Bim, Apoptosis ↓Bcl-xl, Mcl-1, XIAP, HDAC7	[141]
	In vitro	HH, MJ cells	ABT-737 + Panobinostat	↑ Apoptosis, cleaved PARP	[141]
	In vivo	Athymic nude mice (HH cells) xenograft	Panobinostat	↑ Survival ↓ Tumor volume	[141]
PPAR	In vitro	Daudi, Ramos cells	Ciglitazone, Troglitazone, 15d-PGJ2^A^	↑ Apoptosis ↓ Cell viability	[142]
PPARγ	In vitro	HH cells	CDDO^A^ + Bexarotene	↑ Apoptosis	[95]
	In vitro	MJ, Hut78, and HH cells	CDDO^A^	↑ Apoptosis	[95]
	In vitro	SU-DHL6 cells	T0070907^B^, GW9662^B^	↓ Cell growth	[143]
RXR/RAR	In vitro	MJ, HuT78 cells	AM580^A^ (RARα), CD2314^A^ (RARβ), BMS961^A^ (RARγ)	↑ Surface β7 integrin, Cell adhesion, Apoptosis	[144]
	In vitro	MJ cells	ER50891^B^ (RAR*α*)	↓ Cell adhesion	[144]
	In vitro	HuT78, MJ, MyLa, SeAx cells	AM580^A^ (RARα) + Bexarotene^A^ (RXR)	↑ Cell adhesion, Apoptosis, cleaved Caspase-3 ↓ Bcl-2, Survivin, Proliferation	[144]
	In vitro	HuT78, HuT102 cells	AM580^A^ (RARα) + Bexarotene^A^ (RXR)	↑ Chemotaxis towards CCL25	[144]
	In vitro	MJ, Hut78 cells	ATRA, Bexarotene^A^, ATRA + Bexarotene^A^	↑ CCR9^+^ cells%, Chemotaxis towards CCL25	[144]
RXR	In vitro	DT40 (RXR OE) cells	VTP194204^A^	↓ Cell growth	[145]
		DT40 (RXR OE), Jurkat (RXR OE) cells	VTP194204^A^	↑ Caspase-3 & -8 activity, Apoptosis	[145]
	In vitro	MJ, HuT78, HuT102, SeAx cells	Bexarotene^A^ /ATRA + Manganese	↑ *β*7 integrin activation, Cell adhesion	[146]
	In vitro	HuT78 cells	Bexarotene/ATRA	↑ Cell adhesion, CCL25	[146]
	In vitro	HuT78, MJ cells	Bexarotene/ATRA + 1, 25 (OH)_2_ D_3_	↓ Cell adhesion	[146]
	In vitro	HuT78 cells	ECPRIM	↑ Apoptosis, G0/G1 arrest, p21 ↓ Proliferation, pJAK1, pSTAT3, pSTAT5, Cyclin D1, Cyclin E, CDK2, CDK4, Bcl-xl, c-Myc	[147]
	In vitro	MyLa, HuT78 cells	9-cis UAB30^A^, Bexarotene^A^	↑ Apoptosis, p27kip1 protein stability ↓ Cell number, G1 to S cell cycle transition, SKP2 PSMA7	[148]
	Clinical	CTCL patients	Bexarotene, Bexarotene + atorvastatin, Bexarotene + atorvastatin/ fenofibrate, Bexarotene + gemfibrozil	↑ Overall response rate	[149]
	In vitro	U2OS cells	Vorinostat + Bexarotene^A^	↑ RARβ, DR5 ↓ Cell viability	[150]
	Clinical	CTCL patients	Vorinostat + Bexarotene^A^	↑ Overall response rate	[150]
	In vitro	DT40 (RXR OE), Jurkat (RXR OE) cells	AGN194204^A^	↑ Caspase-3, -9 ↓ Cell growth	[151]
	In vitro	Jurkat-pSupCK1*α* cells	AGN194204^A^	↑ Apoptosis, cleaved Caspase-3, -8 ↓ Cell growth	[151]
	Clinical	CTCL patients	Denileukin diftitox + Bexarotene^A^	↑ Overall response rate, CD25	[152]
	Clinical	CTCL patients	Bexarotene^A^	↑ Overall response rate	[153]
VDR	In vitro	HDLM2, L428 cells	Calcipotriol^A^, EB1089^A^	↓ Cell growth	[154]
	In vitro	L428 cells	Calcipotriol^A^, EB1089^A^	↑ nuclear VDR	[154]

**Table 4:** Mechanistic role of various nuclear receptors in Multiple Myeloma in the presence of their agonists/antagonists (^A^Agonist, ^B^Antagonist)

Nuclear receptors (NRs)	In vitro*/*In vivo*/ *In silico/ Clinical	Models/cell lines/condition	Agonist/Antagonist	Results	References
ERβ	In vitro	LP1, CAC-2, CAC-6, NCI H929, U266 cells	4-Hydroxy Tamoxifen^B^	↑ Apoptosis ↓ Proliferation	[155]
	In vitro	LP1, NCI H929 cells	4-Hydroxy Tamoxifen^B^	↑ G1 Arrest, Apoptosis ↓ Proliferation	[155]
GR	In vitro	OPM-1, OPM-2 cells	Dex^A^	↓ Living cells	[156]
	In vitro	MM.1S cells	Trametinib, Dex^A^	↑ Apoptosis, cleaved PARP, BIM ↓ Mcl-1, FAK, PYK2, pPYK2, FLT3, pFLT3, pNDRG1, p4EBP1, pERK1/2	[157]
	In vivo	NSG mice (MM.1S tumor xenograft model)	Trametinib, Dex^A^	↓ Tumor growth	[157]
	In vitro	MM.1S, MM.1R cells	Triptolide	↑ Apoptosis, p-GR, cleaved PARP, cleaved caspase-3 ↓Cellular viability	[158]
	In vitro	MM.1S, MM.1R cells	Triptolide, Triptolide + Dex^A^	↓ Cellular viability	[158]
	In vitro	CEM-S2, CEM-R8, MM.1S, and MM.1R cells	Dex^A^, Rolipram, Forskolin	↑ BimEL, ↓ cell viability, GR, p-Bad	[159]
	In vivo	Nude mice (RPMI-8226 xenograft)	3'-substituted (Z)-5-(2'-(thienylmethylidene))1,2-dihydro-9-hydroxy-10-methoxy-2,2,4-trimethyl-5H-chromeno[3,4-f] quinolines^A^	↓ Tumor volume	[160]
	In vitro	MM.1S cells	miR-130b (OE)	↓GR, GILZ, cleaved PARP	[161]
	In vitro	OPM1, OPM2 cells	Dex^A^	↑ GR mRNA ↓ Immunoglobulin (Ig-*λ*)	[162]
	In vitro	OPM1, RPMI-8226 cells	Dex^A^	↑ GR mRNA ↓ Cell growth	[162]
	In vitro	MM.1S cells	Dex^A^	↓ Cell survival, GR, Cell viability, IL-23A, SKP2, BUB1, SREBF1, ↑GILZ, miR-150-5p, FKBP5	[163]
	In vitro	MM.1S cells	CpdA + Dex^A^	↑ cleaved Caspase-3, -7, cleaved PARP, ↓ Cell viability	[164]
	In vitro	MM.1S cells	CpdA	↑ HSP 70, cleaved PARP, ↓ Cell viability	[164]
	Clinical	MM patients	Prednisone	Overall response rate of 10% was observed	[165]
	In vitro	MM1R cells	GR OE + Dex^A^	↑ Apoptosis, p-RAFTK, BIM_L_, BIM_S_,	[166]
	In vitro	MM1R cells	GR mutants OE (S425G, L436V, N454/A458T) + Dex^A^	↑ Apoptosis	[166]
	In vitro	Opm-2, RPMI-8226, ANBL-6 cells	Dex^A^	↑ Apoptosis ↓ NF-κB transrepression	[166]
PPAR	In vitro	MM cell lines (RPMI-8226 and U266), BMSCs, (HS-5)	PPAR*γ* OE	↑ Apoptosis, G2/M phase arrest, cleaved PARP, cleaved Caspase-3 ↓ Cell proliferation, Cell viability	[167]
	In vitro	ANBL6, RPMI-8226 cells	15d-PGJ2^A^, Ciglitazone^A^	↑ Apoptosis, Caspase-2, -3 ↓ Cell viability	[168]
	In vitro	HS-Sultan cells	15d-PGJ2^A^	↑ Apoptosis, cleaved PARP, cleaved Caspase-3, -8, -9 ↓ Cell survival, p-IκBα, NF-κb, cIAP-1, cIAP-2, XIAP, cFLIP	[169]
	In vitro	LP-1, U-266, RPMI-8226-S, OPM-2, IM-9 cells	15d-PGJ2^A^, Rosiglitazone^A^, Pioglitazone^A^	↑ Apoptosis	[170]
	In vitro	U266B1 cells	T0070907^B^, GW9662^B^	↓ Cell growth	[143]
RAR	In vitro	RPMI-8226 cells	ATRA^A^	↑ TGase II, Apoptosis, RARβ, RARγ	[171]
	In vitro	RPMI-8226 cells	CD2425^A^ (RXR) + AM580^A^ (RARα), CD2425^A^ (RXR) + CD417^A^ (RARβ), CD2425^A^ (RXR) + CD437^A^ (RARγ)	↑ TGase II, Apoptosis	[171]
	In vitro	RPMI-8226 cells	CD367^A^ + CD2425^A^ (RXR)	↑ TGase II, Apoptosis	[171]
RXR	In vitro	ANBL6, RPMI-8226 cells	9-cis RA^A^, 9-cis RA^A^ + 15d-PGJ2^A^, Ciglitazone^A^ + 9-cis RA^A^	↓ Cell viability	[168]
	In vitro	RPMI-8226 cell	CD367^A^(RAR) + CD2425^A^	↑ TGase II, Apoptosis	[171]
VDR	Clinical	MM patients	Bortezomib + Dex + lenalidomide/cyclophosphamide	↑ Overall response rate, Progression free survival	[172]

### AR

AR, also known as NR3C4, is a ligand-responsive TF that is a constituent of the NR superfamily. This receptor is situated on the X chromosome at the Xq12 locus [173–175]. In humans, AR expression can be identified in various tissues such as the breast, cervix, endometrium, epididymis, fallopian tube, kidney, seminal vesicle, and testis [176]. AR and its predominant ligands, the androgens, play a pivotal role in shaping the male phenotype and modulating gene expression associated with cellular growth and differentiation in its target organs [177]. A multitude of pathologies, encompassing androgen insensitivity syndrome (AIS), various cancers like those of the prostate, breast, ovary, and pancreas, anabolic conditions like osteoporosis and muscle wasting, are intricately linked to aberrant AR expression [178]. Depending on the specific malady, different AR ligands such as agonists, antagonists, and modulators, are perceived as potential therapeutic strategies, thereby highlighting the significance of AR as a therapeutic nexus [179]. Mostaghel et al., revealed that the treatment of enzalutamide, a potent AR antagonist, inhibited growth of various mantle cell lymphoma (MCL) cell lines examined, regardless of their gender origin (Granta, Jeko-1, RAMOS, Rec-1, Maver-1 cells). This finding suggests that targeting the androgen pathway could represent an innovative therapeutic strategy for addressing MCL [134]. However, further studies in the near future could lead to a better understanding of AR in the treatment and management of HM.

### ER

The ER, known as nuclear receptor subfamily 3, group A (NR3A), serves as a critical TF involved in regulating various intricate physiological mechanisms in humans [174, 180]. Two primary isoforms of ER exist, namely ERα (NR3A1) and ER*β* (NR3A2), each encoded by genes located on chromosomes 6q25.1 and 14q23.2, respectively. Structurally, ERs encompass three functional domains: the NTD, DBD, and CTD [180]. Deregulation in ER signaling pathways can lead to a spectrum of disorders, including metabolic syndromes, cardiovascular ailments, neurodegenerative conditions, inflammation, osteoporosis and cancers [180]. ERα predominantly localizes to tissues like the breast, certain smooth muscle regions, cervix, endometrium, fallopian tube, and vagina, whereas ERβ finds primary expression sites in the adrenal gland, rectum, testis, ovary, lymph node, and tonsil [176]. As a constituent of the NR protein family, ERs are primarily nuclear but can also be localized in the mitochondria and cytoplasm [180]. Estrogen, the primary ligand for ERs, oversees several cellular activities ranging from cell growth and differentiation to reproductive functions. Moreover, estrogen influences skeletal development and maintenance and ensures the optimal functionality of the neurological and cardiovascular systems (https://www.genecards.org/cgi-bin/carddisp.pl?gene=ESR1&keywords=esr1) [180]. Preliminary research also reported that ERβ2 (one of the splice variant of ERβ) expression is elevated in chronic lymphocytic leukemia (CLL) patients relative to healthy counterparts [64].

Nishikawa et al., reported that administering busramustine (KM-2210), a conjugate derived from 17 beta-estradiol and chlorambucil's benzoate, effectively diminishes hepatosplenomegaly, reduces the count of enlarged lymph nodes, lowers peripheral blood lymphocyte levels, and enhances survival outcomes in patients with CLL [67]. Another study revealed that the treatment of a synthetic compound bisphenol A (BPA) or genistein (a dietary phytoestrogen) resulted in the induction of apoptosis (genistein only) and reduced proliferation and tumor growth in vivo, suggesting antiproliferative effects of BPA and genistein on ERβ-expressing lymphomas [137]. Further, another study reported that ERβ-selective agonists diarylpropionitrile (DPN) and KB9520 strongly inhibited proliferation of Ramos and Raji cells [135]. Additionally, induction of apoptosis and reduction in tumor volume was also observed in vivo when treated with DPN and KB9520 [135]. Another in vivo study determined that activating ERβ by its agonist DPN resulted in anticancer activities such as inhibition of angiogenesis, lymphangiogenesis, tumor growth, Ki67, BAFF, and GRB7 expression [136]. Overall, these studies implicate ERβ modulation as a plausible approach for managing and treating lymphomas. An intriguing study demonstrated that the treatment of 4-hydroxy tamoxifen (4-OHTam), a selective estrogen receptor modulator (SERM), decreased proliferation and increased apoptosis in LP1, CAC-2, CAC-6, NCI H929, U266 cells and G1 phase cell cycle arrest in LP1, NCI H929 cells [155]. However, more studies are needed to establish the potential of ER as a therapeutic target for HM.

### ERR

ERR is an orphan member of the NR superfamily. Functioning as a ligand-dependent orphan receptor, ERR is classified into three distinct subtypes: ERRα (NR3B1), ERRβ (NR3B2), and ERRγ (NR3B3). These receptors play a pivotal role in modulating metabolic activities across a variety of tissues, including skeletal muscle, adipose tissue, bone, and liver [174, 181, 182]. ERRα is localized to the chromosomal locus 11q13.1, ERRβ to 14q24.3, and ERRγ to 1q41 [175]. ERRα is ubiquitously expressed throughout the body with notable exceptions in regions such as the oral mucosa, vagina, ovary, smooth muscle, and spleen. In contrast, ERRγ exhibits expression in specific tissues and organs including the stomach, kidney, cerebral cortex, cerebellum, nasopharynx, bronchus, lung, esophagus, colon, rectum, testis, prostate, breast, heart muscle, smooth muscle, and skeletal muscle [176].

A study by Seo W et al. demonstrated a correlation between ERRα expression and the onset and progression of AML. Moreover, leukemic cells exhibited an upregulated expression of ERRα compared to hematopoietic stem and progenitor cells derived from healthy individuals. The same study further indicated that ERRα plays a crucial role in regulating mtOXPHOS in AML cells. Intervention with XCT-790, an inverse agonist for ERRα, or through gene knockdown led to a decrease in mtOXPHOS activity, enhancing antileukemic responses both in vitro and in vivo [65]. However, further investigations into ERR are needed to elucidate its role in HM more comprehensively.

### GR

GR, also known as nuclear receptor subfamily 3, group C, gene 1 (NR3C1), functions as a TF activated by steroid hormones. Upon interacting with various glucocorticoid response elements (GRE), it can either enhance or suppress the transcription of genes integral to processes such as development, metabolism, and inflammatory responses. This receptor encompasses two distinct domains designated for engagement with glucocorticoids, coregulators, and DNA response elements. Additionally, it features a large intrinsically disordered segment that facilitates condensate formation [174, 183].

Alternative splicing and alternative translation initiation can produce different GR isoforms with distinct functions [184]. By undergoing alternative splicing at the 3ʹ end of the NR3C1 gene, two receptor isoforms, GRα and GRβ, are formed, and they have variations in their extreme C-terminal regions [185]. The GRα isoform protein encodes the prototypic functionally active receptor. Conversely, the GRβ splice variant employs an alternate splice acceptor locus within exon 9, resulting in a shorter protein that has a unique 15-amino acid sequence at its C-terminal end [185]. The unique sequence of the GRβ variant impedes ligand binding, producing an inherent nuclear isoform. This isoform has been reported to function as a dominant-negative antagonist of GRα, influencing genes that are both upregulated and downregulated by GCs. Nevertheless, this claim has been challenged from certain studies [185–191]. Moreover, further studies have elucidated that GRβ possesses the capability to both induce and repress gene transcription, independent of GRα transcriptional activity [185, 190]. Elevated levels of GRβ were detected in a single case of GC-resistant CLL. Furthermore, diminished GRα:GRβ mRNA expression ratios have been associated with decreased susceptibility to GC-induced apoptosis in pediatric ALL [185, 192, 193].

GR is ubiquitously distributed across the body and is situated on chromosome 5 at the q31.3 locus [175, 176]. This receptor manifests a dual role mechanism, acting as a TF that binds to GRE, found in both mitochondrial and nuclear DNA, and as a modulator of other TF. GR influences cellular processes such as proliferation, differentiation, and inflammation in target tissues and also plays a role in chromatin remodeling [194].

Shipman et al. identified that dexamethasone (Dex)  administration reduced GR site availability in leukemia patients. Thus, for accurate quantification of GR sites in these individuals, this study suggestedto avoid glucocorticoids for a span of three weeks before receptor analysis [69]. Another study indicated that Dex administration to mice with primary T lineage acute lymphoblastic leukemias (T-ALLs) enhanced survival rates, an effect further amplified by the addition of GDC0941, a pan-PI3 kinase inhibitor. However, sustained treatments led to the rise of drug-resistant variants, with nearly 30% of these variants exhibiting diminished GR protein expression [70]. Further, the treatment of 2-(4-acetoxyphenyl)-2-chloro-N-methylethylammonium-chloride (CpdA) on CEM cells inhibited cell proliferation by targeting the NF-κB pathway [68]. In a different approach, the Traditional Chinese Medicine, Huai Qi Huang (HQH), was found to amplify the sensitivity of ALL cells to Dex or PD98059 (a MEK inhibitor) by elevating GRα levels and inhibiting the MEK/ERK pathway. The combined treatment of HQH and Dex or PD98059 on Jurkat and Nalm-6 cells elevated the levels of pro-apoptotic markers, establishing a potential treatment paradigm for leukemia [71]. Furthermore, an in vitro study revealed that rolipram, a specific phosphodiesterase-4 (PDE4) inhibitor, increased GRα expression. When combined with glucocorticoids like Dex, it amplified the rate of apoptosis in leukemic cells [73]. Another study showcased that either independent or combined treatment of rapamycin with Dex induced apoptosis, prompted cell cycle arrest, and increased GRα and caspase-3 activity while suppressing cell proliferation and mTOR expressions in CEM-C1 cells [72].

Therefore, these strategies could be potentially exploited to provide therapeutic benefits for leukemia patients.

Lesovaya et al. compared the action of CpdA in human T- and B-lymphoma cells expressing GR and their counterparts with silenced GR [68]. It was observed that CpdA effectively inhibited the growth and viability of these cancer cells in a GR-dependent manner. Additionally, it was also demonstrated that a significant association between CpdA and bortezomib aids in inhibiting the growth and survival of T- and B-lymphoma cells, which was found to be highly dependent on GR. Hence, these findings provide a solid basis for developing a new therapeutic approach for HM that combines selective GR agonists (SEGRAs) and proteasome inhibitors [68]. Another study reported that treatment with Dex decreased GR sites in lymphoma patients, suggesting refraining from administering any glucocorticoids before determining receptor numbers [69].

Sriskandarajah et al. (2020) revealed that trametinib (Tra) and Dex combination treatment exhibited antiproliferative properties in RAS-mutant MM cell lines by inhibiting pro-survival PDK1 signaling and activation of apoptotic pathways. Additionally in vivo study revealed that Tra-Dex combination treatment significantly inhibited tumor growth [157]. Multiple studies have reported that the treatment of Dex alone or in combination with CpdA / rolipram, forskolin, triptolide has resulted in multiple anticancer activities, such as upregulation in the expression of GR, PPAR*γ,* increase in the rate of apoptosis and decreased cell survival, cell viability and reduced immunoglobulin λ (Ig-*λ*) levelsetc. [156, 158, 159, 162–164, 166]. Furthermore, an in vivo study showed that 3'-substituted (Z)-5-(2'-(thienylmethylidene))1,2-dihydro-9-hydroxy-10-methoxy-2,2,4-trimethyl-5H-chromeno[3, 4-f] quinolines resulted in reduced tumor volume in mouse xenograft models [160]. However, more studies in the future could strengthen the grasp of GR in the treatment and management of HM.

### LXR

Liver X Receptors (LXR), classified as NR1H, and are subdivided into LXRα (NR1H3) and LXRβ (NR1H2), positioned on chromosomes 11p11.2 and 19q13.3, respectively [174, 175]. LXRs are crucial for lipid and cholesterol metabolism and also modulate various inflammatory pathways. These receptors are  potential therapeutic targets in diverse diseases, encompassing neurological disorders, metabolic diseases, and even cancer [195]. Structurally, LXR proteins comprise a zinc-finger DNA-binding domain and a lipophilic ligand-binding domain. Ligand association triggers a structural rearrangement in LXR, facilitating coactivator binding and subsequent target gene transcription [196, 197]. These isoforms maintain about 77% sequence similarity [198]. LXRα displays ubiquitous expression throughout the body, while LXRβ exhibits its expression in bronchus, esophagus, testis, placenta, skeletal muscle, skin, cerebral cortex, cerebellum, adrenal gland, oral mucosa, stomach, small intestine, colon, rectum, gallbladder, pancreas, kidney, urinary bladder, epididymis, vagina, fallopian tube, cervix and tonsil [176]. Functionally, LXRs collaborate with RXRs to form heterodimers, with ligands for either receptors potentially activating the complexes [198]. Activation of LXR/FXR heterodimers by agonists governs the expression of a wide array of genes [196]. Acting as cholesterol homeostasis sensors, LXRs promote cholesterol efflux while suppressing its influx and synthesis in response to increased intracellular cholesterol under physiological conditions. Elevated intracellular cholesterol accompanies both normal and cancer cell growth. With diminishing intracellular oxysterol concentration leading to LXR activation, this highlights LXR's role in cancer therapy, potentially through cholesterol metabolic pathways [198].

Recent investigations have identified that LXRs potentially modulate cell growth and viability in CLL patients [196]. Another study reported the effects of dendrogenin A (DDA), a cholesterol derivative known for its tumor-inhibiting attributes and as a partial LXR agonist. Upon leukemic cell exposure to DDA, it resulted in the induction of autophagy. This treatment also stimulated the expression of Nor1 and Nur77, leading to the formation of autolysosomes while concurrently suppressing 3β-hydroxysterol-Δ8,7-isomerase (D8D7I), inducing sterol accumulation in both in vitro and in vivo [74]. Further, when DDA was combined with daunorubicin and idarubicin, there was a collaborative increase in cell mortality. Moreover an increased rate of DNA damage was observed when treated with idarubicin alone in AML cells in an LXRβ-dependent manner [75].

A study by Ceroi et al., proved that treatment of CAL-1 and GEN2.2 cells with T0901317 and GW3965, two LXR agonists, caused apoptosis and elevated the expression of LXR, ABCG1, and ABCA1 genes. Further, these compounds suppressed cell proliferation via the NF-κB pathway. Furthermore, it was also observed that T0901317 prevented cytopenia and blastic plasmacytoid dendritic cell neoplasm (BPDCN) cell infiltration in vivo [139]. The expression studies of LXR in HM is limited. Therefore, more studies could lead to a better understanding of LXR's role in HM.

### Nur77

The NR4A receptor is a member of the orphan NR family, encompassing Nur77 (NR4A1), Nurr1 (NR4A2), and Nor1 (NR4A3). These receptors typically activate transiently, orchestrating differential activation of NR4A-responsive genes that govern diverse biological functions and pathological conditions such as cell cycle, DNA restoration, inflammation, metabolism, apoptosis, atherogenesis, and oncogenesis [174, 199]. Notably, in AML mice models, both NR4A1 and NR4A3 have been identified as tumor suppressors [199]. The chromosome 12q13.13 houses the Nur77 gene [175]. Beyond specific tissues like the parathyroid, liver, prostate, spleen, bone marrow, and adipose, Nur77 exhibits a wide physiological distribution [176]. Nur77 is acclaimed for its significant influence on apoptosis in various cancerous cells [200]. Predominantly, Nur77 functions as an inflammatory modulator. In experimental and clinical settings, its expression is differential in chronically inflamed organs and elevated upon immune cell activation. Further, in vivo investigations concerning inflammatory ailments have discerned that alterations in Nur77 expression can affect the course of the diseases [201]. Structurally, Nur77 comprises an NTD, a LBD, and a DBD [202]. The effects of Nur77 have an influence on cancer cells, with its activation being regulated by its subcellular localization. In the nucleus, Nur77 acts as an oncogenic survival factor and promotes the proliferation of cancer cells [203]. Numerous studies indicate that Nur77 aids in the control of apoptosis in distinct cancer types [204–206].

Yu et al. (2020) reported that cantharidin, a main medicinal component of Mylabris (blister beetle) treatment caused a reduction in cell viability, colony formation ability, proliferation, induced apoptosis, cell cycle arrest at the G2/M phase and increased Nur77 expression in AML cells (HL-60). Besides, it also demonstrated an antileukemic effect in NOD/SCID mice with the injection of HL-60 cells into the tail vein [78]. Another study reported that in both AML cells and CD34^(+)^/38^(−)^ AML LSCs, the inhibition of HDAC using a class I HDAC inhibitor SNDX-275 restored the expression of Nur77/Nor1 and induced the expression of activator protein 1, c-Jun and JunB as well as the death receptor TRAIL in both AML cells and CD34^(+)^/38^(−)^ AML LSCs. Further, SNDX-275 increased the transcription of the pro-apoptotic proteins Bim and Noxa in LSC and AML cells [76]. Another study revealed that the treatment of HL-60 cells with AZA and metacept-1 (MCT-1) alone or in combination resulted in the inhibition of cell growth, MMP-9, and Bcl-xl expression along with the induction of apoptosis, caspase-3, p15^INK4b^, and p21^WAF1/CIP1^ expression [79]. Another study demonstrated that Z-ligustilide (Z-LIG), the main phthalide of *Rhizoma chuanxiong*, caused inhibition in the rate of proliferation and colony formation ability along with a concentration-dependent effect on apoptosis induction and restoration of Nur77 and Nor1 expression in AML cells by increasing the expression of Ace-H3. Moreover, in NOD/SCID mice, silencing of Nur77 and Nor1 reduced the antiAML activity of Z-LIG, suggesting Z-LIG has the potential to function as a new bifunctional agent in the treatment of AML, as it has the ability to restore both apoptosis and differentiation mediated by Nur77/Nor1 [77]. Wang et al. revealed that ginsenoside 20(S)-Rh2 triggered Nur77 expression along with antileukemic activities such as apoptosis, differentiation, and expression of the death receptor proteins like Fas, FasL, DR5, and TRAIL, as well as cleaved caspase-3 and -8 in both in vitro and in vivo [80].

An intriguing study unveiled that the overexpression of Nur77 has shown an increase in apoptosis via the upregulation of Bim, Puma, TRAIL and a reduction in tumor growth in vitro and in vivo*.* Subsequently, it was also observed that the treatment of lymphoma cell lines and immortalized B cells with cytosporone B (CsnB), a binding agonist of Nur77, induced Nur77-mediated apoptosis [140]. Another study demonstrated that the treatment of panobinostat alone or in combination with ABT-737 resulted in the induction of apoptosis and elevated expression of Nur77 and Nor1 in HH and MJ cells. Subsequently, it was observed that the treatment of HH xenografts with panobinostat reduced tumor growth and increased the survival rate [141]. In brief, Nur77 plays numerous anticancer activities, such as induction of apoptosis and pro-apoptotic proteins, inhibition of proliferation, cell/tumor growth, etc., suggesting Nur77 plays an important role in the treatment of HM.

### Nor1

Nor1, an integral component of the NR4A receptor family, demonstrates ubiquitous expression in the human body, with notable concentrations in the thyroid and renal systems [176, 207]. Nor1 is located in the 9q22 position in the chromosome [175]. Experimental evidence indicates that when adrenal fasciculata cells are subjected to adrenocorticotropic hormone (ACTH) or angiotensin II, there's an upregulation of Nor1 expression [207]. Detailed temporal analysis of ACTH or angiotensin II interventions suggests that Nor1 functions as an intermediary, influencing the steroidogenic capacities of adrenal cells in response to these hormones. Intriguingly, both Nor1 and NGFI-B/Nur77 display a considerable overlap in their amino acid sequence homology and transactivation characteristics, signifying their parallel structural and functional organization [207]. Nor1 plays a critical role in ensuring cellular homeostasis and in certain pathological conditions. Moreover, it has been identified to posses anticancer attributes across multiple cancer types [208].

An intriguing study reported that SNDX-275 (entinostat) induced the expression of TRAIL, Nur77/Nor1, and pro-apoptotic proteins Bim and Noxa in LSC and AML cells [76]. Another study demonstrated that Z-LIG targets AML strictly by increasing Nur77 and Nor1 mediated apoptosis and decreasing proliferation and colony formation in these cells. Moreover, silencing Nur77/Nor1 in vivo reduced the antiAML activity in NOD/SCID mouse [77]. However, more studies on Nor1 could promote greater insight into the treatment and management of HM.

### PPARs

PPARs, belonging to the NR1C subfamily, are ligand-inducible receptors. Upon activation, they form heterodimers with RXR and migrate to the nucleus, modulating the expression of target genes linked with fatty acid oxidation, glucose and lipid management, inflammation, proliferation, and differentiation via interaction with PPAR response elements (PPRE) [52, 209–211]. The three PPAR isoforms, PPARα (NR1C1), PPARδ (NR1C2), and PPARγ (NR1C3), are chromosomally mapped to 22q13.31, 6p21.31, and 3p25.2 loci respectively, and exhibit significant sequence similarities [175]. While PPARα upregulates genes involved in the fatty acid oxidation cascade, PPARδ modulates fatty acid metabolic processes, and PPARγ enhances fatty acid uptake [209]. Structurally, PPARs consist of four regions: an AF-1 domain that doesn't rely on ligands, a DBD with zinc fingers specific for PPRE, a hinge sector, and a domain for ligand-binding and dimerization [210, 212]. PPARα is expressed in the heart, liver, kidneys, and muscles. PPARγ expressed in adipose tissues, liver, and skeletal muscles and PPARδ is expressed ubiquitously [176]. They recognize and bind diverse ligands, ranging from endogenous entities like fatty acids and eicosanoids to synthetic agents like fibrates and thiazolidinediones, and even diet-derived molecules like carotenoids and polyphenols [213, 214]. They play an important role in several signaling pathways, including oxidative, inflammatory, AMPK, sirtuins, and mTOR. Additionally, PPARs exhibit potential therapeutic impacts across diverse health conditions such as atherosclerosis, inflammation, metabolic disorders, neurodegenerative diseases, and cancers [215].

PPAR activation with agonists such as troglitazone, 2-cyano-3,12-dioxooleana-1,9-dien-28-oic acid (CDDO), TZD18, 15d-PGJ2 and rosiglitazone resulted in the apoptosis of the leukemic cells [87, 88, 90–96, 98, 100–102]. In addition, treatment of the leukemic cell lines with troglitazone alone or in combination with 15d-PGJ2 resulted in enhanced apoptosis and G1 cell cycle arrest, accompanied by an increased expression in cleaved caspase-3, Bax, and monocyte differentiation as well as decreased cell proliferation, c-myc, survivin, Bcl-2, COX-2, Tcf4, and pRb levels [81–84, 99]. Another study has reported that leukemic cell lines treated with thiazolidinediones caused a reduction in cell proliferation by decreasing Bcl-2 expression along with escalated G_0_/G_1_ phase cell cycle arrest and monocytic differentiation by increasing the expression of  Bax, Bcl-xl, CD11b and CD14 [89]. Further, treatment of pioglitazone in combination with imatinib resulted in the inhibition of STAT5 and its target genes, such as Bcl-xl, Bcl-2, proviral integration site for moloney murine leukemia virus-1 (PIM), and cytokine inducible SH2- containing protein (CIS) and increase in OCT1 expression [106]. A couple of studies showed that treatment of THP-1 cells with rosiglitazone and 9-cis RA caused the suppression of growth and migration of these cells [104, 105].

Several studies have reported that leukemic cells, when treated with CDDO, resulted in the activation of PPAR and induction of apoptosis. However, treatment with PPAR antagonists was unable to reverse the antiproliferative effects [88, 92, 94, 95, 97, 98, 103]. Another study revealed that the treatment of leukemic cell lines with 15d-PGJ2 resulted in the induction of TRAIL expression and apoptosis [86]. These results suggested that 15d-PGJ2 and CDDO despite being PPAR agonists, exerted their antiproliferative efforts in a PPAR-independent manner.

Numerous studies have revealed that treatment of rosiglitazone, pioglitazone, T0070907, and GW9662 on lymphoma cell lines inhibited cell growth or proliferation and induced apoptosis [142, 143].

An accumulating number of studies showed that the treatment of myeloma cell lines with 15d-PGJ2 and thiazolidinediones (rosiglitazone, pioglitazone, ciglitazone, and triglitazone) either alone or in combination resulted in induction of apoptosis by increased mitochondrial depolarization, and caspases activation, as well as a decrease in cell adhesion, IL-6 production, and NF-κB-dependent antiapoptotic proteins [143, 168–170]. In addition, ectopic expression of PPARγ resulted in increased cell death and decreased cell proliferation accompanied by inhibition of IL-6 production in myeloma cells [167].

### RARs

RAR, classified under the nuclear receptor subfamily 1, group B (NR1B) category, is a member of the NR superfamily [174]. Serving as the receptors for retinoids, which are structurally and functionally analogous to vitamin A, RARs modulate numerous vital cellular processes and play a protective role against carcinogenesis. Both RARs and RXRs function as central mediators in the retinoid pathway, and each features three distinct subtypes that further diversify into multiple isoforms [216]. RAR encompasses three isoforms: RARα (NR1B1), RARβ (NR1B2), and RARγ (NR1B3), localized at chromosomal positions 17q21.2, 3p24.2, and 12q13.13 respectively [175, 217]. While RARα and RARγ exhibit a broad expression pattern across various tissues, RARβ's expression is more restricted, being absent in several organs and tissues such as the parathyroid gland, liver, and kidney among others [176]. In their active form, RARs operate as ligand-sensitive TF, often associating with RXR members in a heterodimeric arrangement to interact with specific retinoic acid response elements (RAREs) within the promoter region of target genes. Additionally, RARs can activate kinase signaling cascades and exhibit non-genomic activities that refines the expression patterns of retinoic acid (RA) responsive genes. Disturbances in RA signaling cascades are hypothesized to be linked to numerous HM as well as non-HM pathologies, including a diverse array of cancers such as breast cancer, glioblastoma, head and neck cancer, leukemia, liver cancer, lung cancer, neuroblastoma, ovarian cancer, pancreatic cancer, prostate cancer, renal cell carcinoma, and skin cancer [217].

A study by Koshiishi et al., revealed that phenyl-thiazolyl-benzoic acid derivative (PTB), a potent agonist of RXRα and RARα exhibited complete activation of reporter genes containing enhancer elements specific to RXRα/RXRα, while it only partially activated reporter genes with enhancer elements for RXRα/RARα, PPARδ(β), and PPARγ. Additionally, PTB induced cell differentiation and effectively suppressed the growth of human APL cells (HL-60, NB4). As a result, PTB appears as a unique dual agonist of RXRα and RARα, acting in leukemic cells as both an inducer of cell differentiation and a suppressor of cell proliferation [107]. In a clinical study, it was reported that the use of ATRA and arsenic trioxide (ATO) as a treatment regimen exhibited notable improvements in APL patients. These improvements included an increased rate of complete remission, enhanced event-free survival, improved disease-free survival, and a reduced probability of relapse [109]. Another study reported that the treatment with RAR agonists such as ATRA, 9-cis RA, and BMS753 led to the maturation of NB4 cells (APL cell line), however, it was independent of RAR [108]. Further, another study exhibited that the treatment of retinoids resulted in increased RARα expression levels and differentiation rate in HL-60 cells [110]. An accumulating number of studies have reported that the treatment of ATRA in combination with either APO, ATO, and DAC resulted in numerous anticancer activities, such as increased complete remission rate, overall survival rate, disease-free survival rate, RARβ, p16 expression in both pre-clinical and clinical studies [111–114].

Joseph et al. demonstrated that the treatment of ATRA in combination with RAR agonists CD367 and CD2425 resulted in the induction of transglutaminase II (TGase II), RARα, RARβ, RARγ, RXRα, RXRβ, RXRγ expression and apoptosis in RPMI-8226 cells [171]. To conclude, it is evident that RAR plays a crucial role in the modulation of HM upon interactions with various agonists and antagonists. Hence, targeting and altering RAR could further lead to a better understanding for the treatment of HM.

### RXRs

RXRs are members of the NR subfamily NR2B and exist in three distinct isoforms: RXRα (NR2B1), RXRβ (NR2B2), and RXRγ (NR2B3) [174]. Chromosomally, they are situated at 9q34.2, 6p21.32, and 1q23.3 respectively [175]. Both RXRα and RXRβ demonstrate widespread expression across various tissues, whereas RXRγ lacks expression in certain areas, including the tonsils and bone marrow [176]. When activated by their ligands, such as ATRA, RARs form heterodimers with RXRs, influencing gene expression across diverse biological processes [218]. Notably, RXRs form heterodimer partnerships in two classes: the "permissive" group (e.g., PPAR/RXR, LXR/RXR, FXR/RXR) and the "non-permissive" group (e.g., RAR/RXR, VDR/RXR, TR/RXR) [219]. In the former class, RXR ligands can independently initiate transcriptional activity. The precise interaction dynamics between ligand-receptor assemblies and coregulators vary depending on the cellular context, orchestrating specific gene transcriptional patterns [219]. Therapeutically, RXR modulators have been identified as valuable agents for managing a spectrum of conditions, ranging from malignancies to metabolic disorders [219]. Given that NRs principally modulate gene transcription governing cellular functions, they represent attractive therapeutic targets for diverse ailments. Within this intricate regulatory framework, RXR emerges as a key player [220, 221]. The elucidation of RXR and its associated ligand pioneered two seminal theories in NR research. Firstly, it substantiated the existence of a novel signaling cascade, enhancing extensive exploration into orphan receptors and their specific ligands. Secondly, it highlights the propensity of RXR to heterodimerize with these newly recognized orphan receptors, illuminating a network of interwoven signaling routes [222]. Strategizing around RXR activity modulation has been highlighted as a prospective avenue for influencing cellular processes implicated in various conditions, including neurodegenerative disorders like Alzheimer's and Parkinson's disease, metabolic disturbances, and cancers [220]. In the context of HM, Sana et al. observed diminished RXR expression in patient blood samples relative to healthy controls [60].

A clinical study with leukemic patients demonstrated that bexarotene treatment (RXR agonist) in 27 patients (100%) resulted in a reduction in bone marrow blasts (15%), an increase in platelet (41%), and neutrophil count (26%). Moreover, bexarotene contributed to the survival of three patients with relapsed AML for over an year, suggesting the antileukemic activity of bexarotene [115]. An in vitro study revealed that the treatment of  RA resulted in the induction of RARα, RXR, extracellular signal-regulated kinase (ERK2), myeloid differentiation and G_1_/G_0_ arrest in blr-1 OE HL-60 cells [116]. A plethora of studies have reported that RXR ligands such as 9-cis RA, 13-cis RA, SR11278, SR11345, SR11276, SR11236, SR11246, SR11249, SR11256, LGD1069, differanisole A and ATRA alone or in combination resulted in multiple anticancer activities such as increase in cell differentiation, cell cycle arrest, upregulation of RAR, RXR, CD11b expression and decrease in colony formation, proliferation, etc. in human leukemic cell lines [117–122, 124, 125]. In another study, PTB, a potent RXR agonist showed inhibition of cell growth, proliferation, and differentiation of APL cells [107].

Several studies have reported that the activation of RXR by selective agonists such as 9-cis UAB30, bexarotene, ATRA, and AGN194204 alone or in combination has shown anticancer activities. For example, these agents caused induction of apoptosis, p21 expression, cell cycle arrest and inhibition of proliferation, cell growth, etc., in human lymphoma cell lines [144, 148, 151]. Further, another study proved the antiproliferative effects of the second-generation RXR agonist, VTP194204 [145]. Another study depicted that treatment of bexarotene in combination with ATRA resulted in an increased *β* integrin 7 expression besides the study also showed an increased cell adhesion upon treatment with bexarotene combined with Mn^2+^ in human lymphoma cells, MJ, Hut78, Hut102, and SeAx. Additionally, it was observed that treatment of RXR agonist SR11237 and RAR agonist TTNPB resulted in the elevation of cell adhesion [146]. In another study, ECPRIM, an RA derivative, was shown to suppress cell proliferation and antiapoptotic proteins and induce apoptosis and cell cycle arrest [147]. Furthermore, many clinical studies reported that bexarotene alone or in combination with vorinostat, denileukin diftitox, or atorvastatin improved the overall response rate in cutaneous T-cell lymphoma (CTCL) patients [149, 150, 152, 153].

Ray et al. reported that treatment of 9-cis RA alone or in combination with PPARγ ligands such as 15d-PGJ2 and ciglitazone led to induction of apoptosis and reduction in the rate of cell viability in ANBL6, RPMI-8226 cells [168]. Another study demonstrated that the combination of retinoid CD367 and RXR selective agonist CD2425 induced enzyme TGase II. Additionally, when used with ATRA, CD367 partially suppressed the ATRA-induced TGase II, while CD2425 augmented it. Further, apoptosis was observed in RPMI 8226 cells following treatment with ATRA alone or in  combination with CD367 and CD2425, but not when treated with CD367 or CD2425 alone. Moreover, prominent accumulation of TGase II immunoreactivity was also observed in apoptotic cells [171]. Taken together, these studies suggest that RXR plays a vital role in the management of HM.

### SHP

SHP denoted as NR0B2, distinguishes itself within the NR superfamily due to its unique structural features. While it possesses the dimerization and presumptive LBD domains, it lacks the conventional DBD. Notably, SHP can engage in direct interactions with a multitude of NRs, delineating its significant role as a transcriptional repressor in gene expression modulation. Several interacting partners of SHP have been identified, with these interactions impacting a diverse set of genes across multiple biological pathways [174, 223]. Functionally, SHP is involved in mediating the transcription of various target genes pivotal to maintaining the metabolic homeostasis [224]. Genomically, the human SHP gene resides on chromosome 1 at the 1p36.1 locus. Its structural organization comprises two exons interspersed with an intron, which spans approximately 1.8 kilobases in the human [225]. SHP expression is not restricted to a single tissue type. In mice, its primary expression locales are the liver and gallbladder. However, traces of SHP can also be detected in the adrenal glands, brainstem, cerebellum, and several parts of the gastrointestinal system, including the colon and various segments of the small intestine. Other sites of expression include the heart, kidneys, reproductive organs, and pancreas [226]. Similarly, in humans, SHP mRNA has been identified in organs like the adrenal glands, heart, liver, pancreas, small intestine, spleen, and stomach [223]. As a pleiotropic regulator, SHP orchestrates the expression of numerous target genes. These genes play roles in diverse biological processes, encompassing cell cycle regulation, metabolic pathway modulation, stress and inflammatory responses, detoxification processes, cell adhesion and differentiation [227].

Multiple studies revealed that when SHP specific ligands, 5-Cl-AHPN & 3-Cl-AHPC used alone or in combination, decreased cell proliferation and increased cell cycle arrest, and apoptosis in KG-1 AML cells [127, 128]. Kim et al.  (2009) reported that treatment of leukemic (U937) cells with differentiation agents such as phorbol esters (PMA) have increased the levels of SHP, p21^WAF1^, and p65 of NF-κB subunits, suggesting how the expression of SHP increases the cellular survival of differentiating monocytes by transcriptional regulation of target genes of cell survival and differentiation [126]. In conclusion, SHPs play a vital role in tumorigenesis by modulating apoptosis, differentiation, and proliferation of tumor cells. However, further studies could result in a plausible approach to the treatment and management of leukemia.

### VDR

VDR, also known as the calcitriol receptor or NR1I1, is a nuclear TF reliant on ligand activation. Upon binding to the active form of vitamin D, 1,25(OH)2D3 (1,25D3), it orchestrates the regulation of approximately 900 genes implicated in various biological processes [174, 228, 229]. Genomically, VDR resides at the 12q13.11 locus on the chromosome [175]. Additionally, this receptor recognizes lithocholic acid, a secondary bile acid. While VDR oversees an array of metabolic pathways, inclusive of those pivotal for immune response and oncogenesis, its downstream target genes are associated with mineral metabolism (NCBI: https://www.ncbi.nlm.nih.gov/gene/7421). Further, the receptor's association with diverse pathologies, such as cancer, diabetes, and cardiovascular ailments, has been documented [230]. VDR's interaction with 1,25(OH)2D modulates several physiological mechanisms, notably calcium balance and metabolic processes [231–233]. In terms of expression, VDR mRNA is ubiquitously distributed across various tissues, including but not limited to, bone, breast, colon, kidney, lungs, and various immune and endocrine cells. Notably, the parathyroid gland and duodenum exhibit the most pronounced levels of VDR mRNA expression [176, 232, 233]. With regards to inflammatory bowel diseases (IBDs) such as ulcerative colitis and Crohn's disease, the VDR protein plays a crucial regulatory role, influencing immune modulation, epithelial barrier integrity, and cellular proliferation in the intestine. It's also noteworthy that individuals diagnosed with IBD typically display diminished vitamin D/VDR signaling expression [233]. The protective relationship between vitamin D and oncogenesis risk has gained substantial research interest. The intracellular receptor, VDR, upon binding with its active metabolite 1,25(OH)2D, becomes the focal point for the transcriptional modulation of numerous target genes [229, 232]. Multiple studies have reported the downregulation of VDR expression in HM [59, 60].

An interesting study revealed that treatment of human leukemic cell lines MV-4–11, THP-1, and HL-60 with the active forms of vitamin D, calcitriol, and tacalcitol resulted in morphological changes in these cells and suppressed their proliferation [130]. In a clinical study, it was observed that the treatment of azacitidine (AZA) in patients (diagnosed with myelodysplastic syndrome and secondary oligoblastic AML) with high levels of vitamin D tend to show more overall survival rate when compared to patients with low levels of vitamin D. In addition, it was also reported that treatment of AZA in combination with 1,25D3 or 25D3 resulted in the enhancement of antiproliferative effect in HL-60 and MOLM-13 cell lines [131]. Nachliely et al. revealed that VDR agonists and dimethyl fumarate (DMF), a Nrf2 activator caused synergistic effects in increasing VDR and Nrf 2 expression in vitro. Subsequently, it was also reported that the treatment of PRI-5202 (an analog of 1,25D2) in combination with DMF resulted in decreased tumor growth in vivo [132]. Another study demonstrated that the treatment of GSK3 inhibitor SB415286 in combination with 1,25D3 resulted in various anticancer activities such as increased VDR transcriptional activities, activated JNK pathway, and decreased CD14 expression and colony formation in AML cells. Similar results of an increased survival rate were also observed in vivo [133]. Gharbaran et al. reported that the treatment of calcipotriol and EB1089 (vitamin D3 analog) resulted in decreased cell growth and increased nuclear VDR expression in L428 and HDLM2 cells [154]. It has been reported that the treatment of drugs such as bortezomib and Dex in combination with either lenalidomide or cyclophosphamide resulted in overall elevated response rates in MM patients [172]. However, further studies are required to gain a deeper understanding of VDR's mechanistic role in HM.

## Discussion and conclusion

While there have been notable advancements in research, HM continues to be one of the most persistent oncological challenges globally. Diagnosis and therapy at advanced stages of HM are often associated with diminished patient outcomes and survival prospects. Thus, identifying specific therapeutic targets for HM management is imperative. Notably, certain NRs govern the differentiation of myeloid cells, positioning them as potential therapeutic focal points for myeloid leukemia interventions. The effectiveness of ATRA in addressing APL stands as a monumental evidence of the potential of cancer differentiation therapy. ATRA exerts its effects by guiding leukemic cells towards differentiation or apoptosis via RAR interaction. In this discussion, we explore the interplay of NRs, specifically AR, ER, ERR, GR, LXR, Nur77, Nor1, PPAR, RAR, RXR, SHP, and VDR, and their influence on HM cell behaviors. Interactions between NRs and their agonists or antagonists modulate cellular activities such as proliferation, through intricate signaling pathways, such as STAT, NF-κB, MAPK etc. However, comprehensive understanding of the complexities of the NR landscape in leukemogenesis is imperative.

Nonetheless, NR modulators exhibit certain constraints, such as reduced solubility, necessitating intramuscular injection administration, which consequently confines the deliverable volume and dosage. Notably, in extensive patient cohorts, clinical challenges with these modulators have been documented. Preliminary studies conducted on castrate-resistant prostate cancer using certain modulators as monotherapies over a decade ago demonstrated limited efficacy [52, 234]. Yet, recent research, showed that mifepristone in conjunction with enzalutamide, seeks to elucidate their potential benefits (NCT02012296). Pertaining to PPARs, TZDs molecules like RGZ (Avandia) and pioglitazone (Actos) were synthesized in the 1990s. However, their application in oncology is limited. Despite demonstrating antineoplastic activity across various cancer cell lines and potential chemopreventive attributes, TZDs, and PPARγ activation have exhibited minimal therapeutic impact in clinical studies over the past decade and a half [52, 235]. Currently, there is a lack of detailed and comprehensive literature that explains the importance of targeting NRs in the treatment of HM. Therefore, this review offers a comprehensive insight into the roles of NRs in HM, detailing their expression profiles, spatial distribution, molecular interplay, and functional pathways.

Further, modulating these receptors using particular ligands is pivotal in HM treatment strategies. Numerous clinical investigations are underway to explore the therapeutic promise of small molecules targeting NRs for HM. However, individualized patient-specific NR profiles may be created through genomic analysis, making it much simpler to determine which patient will respond best to a particular NR-based medication. Implementing this approach will greatly enhance the effectiveness and broaden the scope of precision medicine [37]. In conclusion, given the array of approved NRs agonists and antagonists, we could foresee that additional investigations in this field may achieve ground-breaking outcomes by repurposing these drugs and presenting promising prospects for the management and therapy of HM.

## Data Availability

Not available.

## Abbreviations

AA: Amino acid
ACTH: Adrenocorticotropic hormone
AF 1: Activation function 1
AMPK: AMP-activated protein kinase
APL: Acute promyelocytic leukemia
AR: Androgen receptor
ATO: Arsenic trioxide
ATRA: All-trans retinoic acid
AZA: Azacitidine
Blr1: Burkitt lymphoma receptor 1
BM: Bone marrow
BPA: Bisphenol A
BPDCN: Blastic plasmacytoid dendritic cell neoplasm
CAR: Constitutive androstane receptor
CART: Chimeric antigen receptor-T cell
CCRL2: C–C chemokine receptor-like 2
CDDO: 2-cyano-3,12-dioxooleana-1,9-dien-28-oic acid; Please add CDDO-Me Methyl-2-cyano-3,12-dioxooleana-1,9(11)-dien-28-oate; Please add CDDO-Im 1-(2-cyano-3,12-dioxooleana-1,9-dien-28-oyl) imidazole
CLL: Chronic lymphocytic leukemia
CML: Chronic myelogenous leukemia
CPDA: 2-(4-Acetoxyphenyl)-2-chloro-N-methylethylammonium-chloride
CsnB: Cytosporone B
CTCL: Cutaneous T-cell lymphoma
D8D7I: 3β-Hydroxysterol-Δ8,7-isomerase
DAC: 5-Aza-2-deoxycytidine
DBD: DNA binding domain
DDA: Dendrogenin A
DEX: Dexamethasone
DMF: Dimethyl fumarate
DPN: Diarylpropionitrile
ERR: Estrogen-related receptor
FDA: Food and Drug Administration
FXR: Farnesoid X receptor
GILZ: Glucocorticoid-induced leucine zipper
GR: Glucocorticoid receptor
GRE: Glucocorticoid response elements
HDAC: Histone deacetylases
HDI: Human Development Index
HM: Hematological malignancy
HNF 4: Hepatocyte nuclear factor 4
HQH: Huai Qi Huang
IBD: Inflammatory bowel disease
Ig-*λ*: Immunoglobulin *λ*
JNK: Jun N-terminal kinase
LBD: Ligand binding domain
LXR: Liver X receptor
MAPK1: Mitogen-activated protein kinase 1
MCL: Mantle cell lymphoma
MR: Mineralocorticoid receptor
NF-κB: Nuclear factor kappa B
NGFI-B: Nerve growth factor IB-like receptors
NR: Nuclear receptor
NTD: N terminal domain
PMA: Phorbol 12-myristate 13-acetate
PPAR: Peroxisome proliferator-activated receptor
PPRE: PPAR response elements
PR: Progesterone receptor
PTB: Phenyl-thiazolyl-benzoic acid derivative
PXR: Pregnane X receptor
RA: Retinoic acid
RAR: Retinoic acid receptor
RARE: Retinoic acid response elements
RXR: Retinoid X receptor
SERM: Selective estrogen receptor modulators
SHP: Small heterodimer partner
TF: Transcription factor
TGase II: Transglutaminase 2
VDR: Vitamin D receptor
Z-LIG: Z-ligustilide
